# Unregulated and Regulated PFASs in Bottled and Tap Water: Occurrence, Co-Occurrence Patterns, and Implications for Human Health and Regulatory Frameworks

**DOI:** 10.3390/jox15030081

**Published:** 2025-05-27

**Authors:** Ioana-Antonia Cimpean, Iuliana Paun, Florinela Pirvu, Vasile Ion Iancu, Florentina Laura Chiriac

**Affiliations:** National Research and Development Institute for Industrial Ecology—ECOIND, Drumul Podu Dambovitei 57-73, Sector 6, 060652 Bucharest, Romania; antonia.cimpean@ecoind.ro (I.-A.C.); florinela.pirvu@incdecoind.ro (F.P.); vasile.iancu@incdecoind.ro (V.I.I.)

**Keywords:** per- and polyfluoroalkyl substances, drinking water quality, environmental contaminants, health risk assessment, analytical methods, public health, environmental persistence, waterborne pollutants

## Abstract

The occurrence of per- and polyfluoroalkyl substances (PFASs) in drinking water remains a critical environmental and public health concern. This study examines the presence of both regulated and unregulated PFASs in twenty-one bottled water and twenty-four tap water samples, assessing their concentrations, co-occurrence patterns, and potential human health implications. Regulated PFASs accounted for 87% of total PFASs in bottled water and 92% in tap water, demonstrating the effectiveness of current EU regulations. However, unregulated PFASs were detected in both water sources, contributing to 13% and 8% of total PFAS concentrations in bottled and tap water, respectively. Principal Component Analysis (PCA) and Pearson correlation matrices revealed distinct clustering patterns, suggesting common contamination sources and potential cumulative exposure risks. The presence of emerging PFASs, such as 4:2 FTSA and HFPO-DA, raises concerns about gaps in regulatory oversight, as their long-term health effects remain largely unknown. Despite EU Directive 2020/2184 setting limits on select PFASs, this study highlights the need for expanded monitoring and stricter regulations to address the full spectrum of PFAS contamination. Given the persistence and bioaccumulative nature of these compounds, a comprehensive human health risk assessment is essential to safeguard public health and ensure the safety of drinking water sources.

## 1. Introduction

Per- and polyfluoroalkyl substances (PFASs) are a class of synthetic chemicals that have garnered significant attention due to their pervasive presence in the environment and potential risk to human health [1,2]. Characterized by their carbon–fluorine bonds, PFASs exhibit unique properties such as heat, water, and oil resistance. They are widely used in various industrial applications and consumer products, including non-stick cookware, waterproof clothing, and food packaging [3,4,5]. Despite their utility, these properties also contribute to PFASs’ high environmental persistence, bioaccumulation potential, and mobility, undergoing negligible degradation in natural systems [6,7,8,9]. While these characteristics are well documented, regional data remain scarce, particularly in Romania and Eastern Europe, limiting our understanding of the local prevalence and human exposure pathways. The emergence of PFASs as contaminants of concern has rapidly evolved into a global public health issue, particularly as emerging research reveals alarming concentrations in drinking water sources worldwide [10,11].

Studies indicate that PFASs have been detected in drinking water supplies across multiple continents, including North America, Europe, and Asia, posing significant risks to public health [10,11,12,13,14,15,16,17]. For instance, recent peer-reviewed studies have revealed that millions of Americans may be exposed to PFASs through contaminated drinking water, prompting increased scientific and regulatory attention to address potential health risks [18,19]. Notably, recent findings have suggested that even trace levels of PFAS exposure can be associated with various health concerns, including immune system impairments, hormone disruption, and an increased risk of certain cancers [20]. These findings underscore the need for comprehensive health risk assessments to inform regulations.

The European Union’s revised Drinking Water Directive (DWD) 2020/2184 establishes two PFAS monitoring parameters: “PFAS total” (all PFAS compounds, limited to 500 ng/L) and the “Sum of PFAS” (20 specific PFAS, limited to 100 ng/L) [21]. Recent technical guidelines published in the Official Journal of the EU defined analytical methods for these parameters [21]. Annex III of the DWD mandates monitoring only if a risk assessment identifies potential PFAS contamination, with compliance required by 12 January 2026 [22]. Experts have raised concerns that the directive does not adequately protect public health, as the 100 ng/L limit is much higher than the European Food Safety Authority’s (EFSA) tolerable weekly intake (TWI) of 4.4 ng/kg body weight per week for four PFASs (PFOA, PFNA, PFHxS, and PFOS), which would correspond to an approximate drinking water limit of 2.2 ng/L [23]. In contrast, the U.S. Environmental Protection Agency (US EPA) recently set Maximum Contaminant Levels (MCLs) for PFASs, including individual limits for PFOA and PFOS at 4 ng/L and PFHxS, PFNA, and GenX at 10 ng/L, along with a Hazard Index MCL for mixtures [24]. Relying solely on theoretical risk assessments may not accurately predict PFAS contamination in specific water sources [25]. The delayed implementation of monitoring requirements until 2026 could further limit the availability of real-time data, leaving suppliers unprepared [21].

The DWD establishes a mandatory baseline for EU Member States to adopt stricter measures. Romania, for instance, introduced Ordinance 7/2023 [26], which governs hazardous substances in drinking water, including PFASs. This aligns with DWD principles and emphasizes preventative measures, water characterization, and PFAS limits, reflecting growing recognition of the need for science-based regulatory oversight.

Despite these advancements, significant gaps remain concerning PFAS concentrations in Romanian drinking water, including differences between bottled and tap sources, geographic distribution, and the resulting human health risks. Moreover, empirical data on the effectiveness of water treatment methods in reducing PFAS levels in the region are limited. To address these issues, this study applied a newly developed LC-MS/MS method to evaluate the presence and concentrations of selected PFASs in bottled and tap water from Romania. A human health risk assessment was also performed to assess the potential implications of PFAS exposure for various age and gender groups.

The novelty of this research lies in applying advanced analytical techniques for PFAS detection and in its focus on a geographic region—Romania and Eastern Europe—for which minimal contamination data exist. Despite increasing awareness of PFAS risks globally, most studies have concentrated on Western Europe or North America, leaving a significant knowledge gap in this part of Europe. By providing region-specific empirical data on PFAS occurrence in tap and bottled water and performing a comprehensive human health risk assessment across demographic groups, this study uniquely contributes to the scientific understanding of PFAS exposure. Furthermore, the results support implementing national policies (such as Ordinance 7/2023) and contribute to international efforts to harmonize regulatory approaches. Our findings underscore the urgency of improved monitoring, regulatory enforcement, and transparent risk communication to uphold the fundamental right to clean drinking water—a cornerstone of public health and environmental justice.

## 2. Materials and Methods

### 2.1. Chemicals and Reagents

The analysis of PFAS compounds was conducted using a variety of high-quality reagents and standards. The EPA Method 533 Native Analyte PDS, which includes 25 analytes, was obtained from Chembridge Isotope Laboratory. The specific PFAS compounds, including perfluorodecane sulfonic acid (PFDS), perfluorooctane sulfonic acid (PFOS), perfluoro octane sulfonamide (PFOSA), and the labeled internal standards perfluorobutanoic acid-^13^C_3_ (PFBA-^13^C_3_) and perfluorooctanoic acid-^13^C_8_ (PFOA-^13^C_8_), were sourced from Sigma-Aldrich (Darmstadt, Germany). The abbreviations and physicochemical parameters for all compounds are listed in Table S1. High-purity solvents were utilized to prepare mobile phases and samples. Methanol of the HiPersolv CHROMANORM^®^ grade, suitable for LC-MS from VWR Chemicals, was employed. An ammonium acetate solution of 5 mM concentration was prepared using deionized water and HiPersolv CHROMANORM^®^ eluents for LC-MS, also obtained from VWR Chemicals. OasisWAX 60μ: PFAS Analysis 6cc 500 mg cartridges from Waters were utilized for sample cleanup and extraction, providing efficient extraction capabilities for the target analytes. All materials were handled and stored following best laboratory practices to ensure the integrity and reliability of the analytical results.

### 2.2. Sample Collection

A total of 45 water samples were collected for analysis, consisting of 24 tap water (TW1–24) samples from various urban and rural households and 21 samples from commercial sources (CW1–21). All household samples were collected in polypropylene bottles to minimize the risk of contamination. Before sample collection, the polypropylene bottles were thoroughly pre-rinsed with methanol and high-purity water to remove potential contaminants. Subsequently, each bottle was rinsed with the respective sample to minimize contamination further and ensure accurate analytical results. To maintain sample integrity, all collected samples were preserved at a temperature of 4 °C during transportation to the laboratory. Samples were analyzed within 48 h of collection to ensure the reliability of the results.

### 2.3. Sample Extraction

The extraction of PFASs from water samples was conducted using Oasis WAX cartridges. Initially, 500 mL water samples were spiked with labeled internal standards to facilitate quantitative analysis of the target analytes. Preparation of the Oasis WAX cartridges involved passing through the following sequential solvents: 4 mL of methanol, followed by 4 mL of high-purity water and another 4 mL of methanol. This conditioning step ensured the cartridges were activated and ready for sample loading. Subsequently, the spiked water samples were loaded onto the cartridges at a flow rate of 5 mL/min to ensure adequate retention of PFAS compounds. After sample loading, the cartridges were washed with 4 mL of a 24 mM ammonium acetate buffer (pH 4) to remove unbound matrices while retaining the target analytes. To further prepare the cartridges for elution, they were briefly dried under nitrogen to remove residual water. Following this step, the cartridges were rewashed with 4 mL of methanol to facilitate elution. The PFAS compounds were then eluted from the cartridges using two consecutive washes with 4 mL of methanol, collecting the eluate in pre-washed 15 mL collection tubes. Following elution, the collected samples were dried under a stream of nitrogen at 50 °C until near dryness to concentrate the extracts. The dried extracts were reconstituted with 0.5 mL of methanol and 0.5 mL of a 4 mM ammonium acetate solution to facilitate analysis. Finally, the reconstituted samples were transferred to polypropylene vials, which were ready for instrumental analysis.

### 2.4. LC-MS/MS Analysis

PFAS compounds were quantified using an Agilent 1260 series liquid chromatography system coupled with an Agilent 6410B triple-quadrupole mass spectrometer (LC-MS/MS) [27]. The chromatographic separation was achieved using an AVANTOR ACE HTP-MS column from the starter kit (contains holder and one column, VWR8), maintained at 30 °C to ensure optimal resolution. A 20 µL injection volume of the reconstituted samples was prepared for analysis. The mobile phase consisted of a 2 mM ammonium acetate solution and 2 mM ammonium acetate in methanol, and the flow rate was set to 0.4 mL/min, in gradient mode. The gradient program is given in Table S2. The samples, originally in water, were processed through a gradient elution program to promote efficient separation of the PFAS analytes over the chromatographic run time of 10.5 min (Table S3, Figure S1). PFAS detection was conducted using multiple reaction monitoring (MRM), allowing for selective and sensitive quantification of the target compounds. The mass spectrometer was operated with a gas flow rate of 9 L/min, and the capillary voltage was set to 1500 V. The operational parameters of the mass spectrometry analysis, including transitions and other relevant settings, are summarized in Table S4. Combining the LC-MS/MS technique with the described conditions ensured high specificity and sensitivity for the detection and quantification of PFASs in drinking water samples.

### 2.5. QA and QC

To guarantee the accuracy and dependability of the data collected for PFAS detection, a comprehensive set of quality assurance and quality control (QA/QC) procedures was diligently applied throughout the sampling, extraction, and analytical stages. During bottled and tap water sampling, field blanks were collected at a frequency of 1 per 10 water samples to monitor potential contamination during the collection process. Before conducting the analysis, the laboratory established baseline reference points, including laboratory blanks, field blanks, spiked samples, and matrix-matched calibration curves to effectively assess the performance of the analytical methods. All samples and reference standards underwent processing under uniform conditions to minimize variability. At the same time, strict procedures were implemented to prevent cross-contamination during the sample collection and laboratory preparation stages. This included using PFAS-free sampling containers, wearing powder-free gloves, and avoiding materials such as PTFE that could leach PFASs. Laboratory blanks and laboratory control samples (LCSs), consisting of known concentrations of PFASs, were inserted at a rate of 1 for every 10 bottled or tap water samples. Method blanks were used to verify the absence of background contamination during processing, while spiked recovery samples were included to assess method performance. Routine LC-MS/MS system maintenance was performed according to the manufacturer’s guidelines, ensuring consistent performance and reproducibility in detecting the target compounds. Spiked samples showed recoveries within 90–110%, and any deviations from these recovery ranges triggered re-analysis or re-injection of the samples. Performance criteria were established, which required that the relative standard deviation (RSD) be kept below 10% for replicate analyses and recovery rates for spiked samples lie between 90% and 110%.

The calibration curves ranged from 0.05 to 0.5 µg/L, achieving correlation coefficients greater than 0.999. The data collected underwent extensive statistical analysis to ensure its robustness, focusing on determining the limits of detection (LOD) and limits of quantification (LOQ). The LOQ was established at 0.06 ng/L, while the LOD was set at 0.02 ng/L, for all targeted compounds. Recovery rates were within a range of 97.5% to 104.8%, with RSD values for inter-day and intra-day tests reported between 3.02% and 4.68%, and 7.37% and 10.2%, respectively, for drinking water samples.

### 2.6. Health Risk Assessment

A health risk assessment was performed to evaluate the potential human health risks associated with exposure to PFASs in drinking water. This assessment estimates the daily intake of PFASs through drinking water consumption and compares it to established tolerable daily intake levels.

The estimated daily intake (EDI) of PFASs, expressed as ng/Kg body weight/day, was calculated using Equation (1):

(1) $$EDI=\frac{\sum \left(Cw\times Dw\right)}{Bw}$$

where

- *Cw* represents the concentration of PFASs measured in surface water.
- *Dw* is the daily volume of drinking water consumed (mL/day).
- *Bw* is the body weight.

For most PFAS compounds, with the exceptions of PFOA, PFNA, PFOS, and PFHxS, the tolerable daily intake (*TDI*) was determined using Equation (2):

(2) $$TDI=\frac{NOAEL}{UF}$$

The *TDI* was established by dividing the observed no adverse effect level (*NOAEL*) by an uncertainty factor (*UF*) of 200. This value incorporates a default factor of 100 to account for interspecies and intraspecies variability, and an additional 2 to address data gaps and uncertainties related to PFAS-specific toxicokinetic and chronic toxicity. This approach mirrors the methodology used by the European Food Safety Authority [28] and was previously applied to determine *TDI* values for substances like PFBS, PFBA, and FTOH [29,30]. In this study, we adopted the same procedure to evaluate the target PFAS, and the calculated TDI data, based on NOAELs [29], are presented in Table S5.

Finally, to characterize the overall risk associated with PFAS exposure from surface water sources used for drinking water, a Risk Index (*RI*) was calculated using Equation (3):

(3) $$RI=\frac{EDI}{TDI}$$

This *RI* indicates the potential health risk by comparing the estimated daily intake to the tolerable daily intake.

## 3. Results

### 3.1. Concentration of Regulated PFASs in Bottled and Tap Water

Per- and polyfluoroalkyl substances (PFASs) in drinking water have emerged as a critical environmental and public health concern due to their persistence, bioaccumulation potential, and associated health risks. Regulated under the EU Drinking Water Directive (DWD) 2020/2184, specific PFAS compounds are monitored to ensure safe drinking water for consumers. This section presents a detailed analysis of the concentrations of regulated PFAS in both bottled and tap water samples (Tables S6 and S7), highlighting key findings and comparisons that elucidate the prevalence of these contaminants in commonly consumed water sources [13]. By assessing the levels of PFASs across different water types, this study seeks to inform stakeholders about potential exposure risks and the need for ongoing monitoring and regulation.

#### Sum of 16 PFASs

The analysis of per- and polyfluoroalkyl substances (PFASs) in bottled and tap water highlights significant differences in contamination levels and distribution patterns, with potential public health implications. The study examined 21 bottled water samples and 24 tap water samples, focusing on regulated PFAS compounds, including perfluoroalkyl carboxylic acids (PFCAs) and perfluoroalkyl sulfonic acids (PFSAs), known for their persistence in the environment.

**Figure 1:**
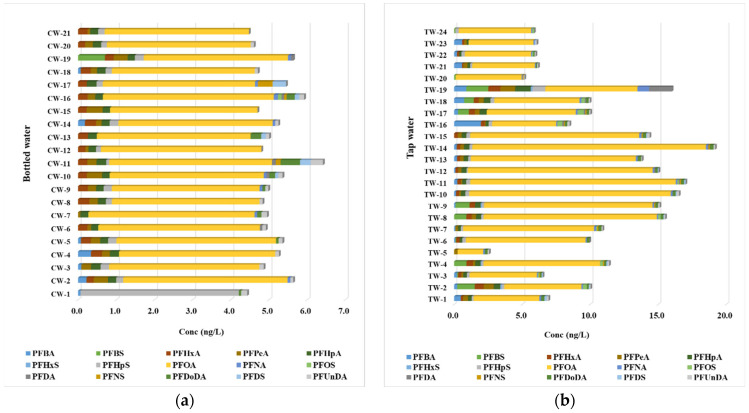
Regulated PFAS levels determined in (**a**) bottled water and (**b**) tap water.

PFAS concentrations in bottled water varied across samples, with PFOA emerging as the most dominant compound (Figure 1a). Several samples, such as CW-1, CW-2, and CW-5, exhibited measurable PFOA levels ranging from 3.75 ng/L to 4.48 ng/L. This finding aligns with previous studies identifying PFOA as a common contaminant due to its widespread historical use in industrial and consumer applications. Additionally, perfluorohexanoic acid (PFHxA) and perfluoroheptanoic acid (PFHpA) were frequently detected in multiple samples, albeit at lower concentrations. For example, PFHxA was observed in CW-2, CW-4, and CW-5, with concentrations ranging from 0.190 ng/L to 0.29 ng/L, while PFHpA was consistently present in CW-3 and CW-5. Despite the prominence of certain PFASs, some compounds, such as perfluorobutanoic acid (PFBA) and perfluorobutane sulfonic acid (PFBS), were either sporadically detected or remained below the limit of quantification (LOQ) in most samples. This suggests that these specific PFASs may be less prevalent in bottled water than others like PFOA or PFHxA. The variability in PFAS concentrations across bottled water samples may be attributed to differences in environmental sources and processing conditions during water treatment and bottling.

Tap water exhibited significantly higher PFAS contamination than bottled water, with greater variability across samples (Figure 1b). PFOA was again the most prevalent contaminant, reaching a maximum concentration of 17.16 ng/L in sample TW-14. Although none of the samples exceeded the parametric values established under Directive (EU) 2020/2184 (e.g., 100 ng/L for the sum of 20 PFAS), the presence of PFOA at this level remains concerning due to its known health effects, including endocrine disruption and developmental toxicity. Other regulated PFAS compounds, such as PFNA and PFOS, were also detected at higher frequencies and concentrations in tap water than in bottled water. Short-chain PFASs, including PFBA and PFBS, were present in multiple samples, with PFBA concentrations ranging up to 1.96 ng/L, indicating localized contamination sources. Interestingly, while PFBS was found at a maximum concentration of 1.28 ng/L, it was detected in fewer samples, reflecting the uneven distribution of PFASs in municipal water supplies. Moreover, long-chain PFASs such as perfluorohexanesulfonic acid (PFHxS) and perfluorododecanoic acid (PFDoDA) displayed lower detection frequencies and concentrations, often falling below the LOQ. From a public health and utility perspective, these findings underscore the importance of targeted monitoring and treatment strategies, especially in areas showing elevated or variable PFAS levels, even when regulatory thresholds are not exceeded.

The summary in Table 1 provides a comprehensive overview of the concentrations of per- and polyfluoroalkyl substances (PFASs) detected in bottled and tap water, facilitating a direct comparison between these two drinking water sources. The data illustrate significant differences in the prevalence and concentrations of various PFAS compounds in bottled water compared to tap water, underscoring diverse potential sources and implications for public health. Regarding PFAS prevalence, PFOA emerges as the most concerning contaminant in bottled and tap water, with bottled water exhibiting a mean concentration of 4.04 ng/L. In comparison, a higher mean concentration of 8.95 ng/L was detected in tap water. This observation is particularly alarming given the strict health advisory thresholds established for PFOA due to its associations with various adverse health effects, including immune system dysfunction and developmental issues. Nearly 100% of tap water samples contained detectable levels of PFOA, compared to a frequency of 95% in bottled water, reflecting that while bottled water is typically perceived as a safer alternative, it can still contain significant levels of harmful contaminants. The analysis also highlights the presence of PFBA and PFHpA in both water types. For PFBA, the maximum concentration in bottled water was 0.34 ng/L, while tap water exhibited a considerably higher maximum of 1.96 ng/L, with frequencies of detection at 33% and 75%, respectively. This disparity suggests that municipal water systems potentially have varied sources of PFBA contamination, which may be linked to industrial discharges or inadequate water treatment processes. Meanwhile, PFHpA demonstrated a similar trend; its maximum concentration in bottled water was 0.26 ng/L, contrasted with a mean concentration of 1.16 ng/L in tap water, confirming again that tap water may expose consumers to relatively higher levels of certain PFASs. Interestingly, PFBS showed a frequency of detection of only 5% in bottled water, suggesting that this compound is not frequently found in this source, whereas in tap water, the detection frequency was significantly higher at 46%. The maximum PFBS concentration in tap water reached 1.62 ng/L, indicating that this compound is more likely to enter municipal water systems than bottled water. This may be attributed to the localized nature of PFBS sources, such as runoff from firefighting foams or industrial activities, preventing contamination of bottled water sources. The analysis of PFHxS revealed that it was not detected in bottled water samples, while showing a low frequency of 17% in tap water. This suggests that PFHxS may have limited distribution in bottled water contexts or indicates effective removal processes during water bottling. However, the presence of PFHxS in tap water raises the necessity for further investigation into the local factors contributing to its occurrence.

The comparative analysis of the sum of per- and polyfluoroalkyl substances (PFASs) in bottled and tap water reveals noteworthy findings that underscore implications for public health in the context of existing European Union regulations. The minimum concentration of total PFASs recorded in bottled water was 4.48 ng/L, whereas tap water exhibited a slightly lower minimum at 2.57 ng/L, suggesting a relatively higher baseline contamination of PFASs in bottled water. However, the maximum concentration noted for tap water (19.2 ng/L) considerably exceeded that of bottled water (6.53 ng/L), indicating that tap water may be subjected to more variable and potentially higher levels of PFAS contamination. The average sum of PFAS concentrations reinforces this observation, with bottled water averaging 5.34 ng/L compared to tap water’s average of 10.8 ng/L. This higher average in tap water highlights potential concerns regarding the quality and safety of municipal water supplies, especially considering the strict regulatory framework established by the Drinking Water Directive (EU 2020/2184) [21]. According to this directive, total PFAS levels in drinking water must not exceed 500 ng/L, and individual PFAS concentrations should not surpass 100 ng/L. Fortunately, both bottled and tap water remain well below these regulatory thresholds. Nevertheless, the markedly higher PFAS levels found in tap water, particularly when considering potential cumulative exposure from various sources, warrant continued monitoring and evaluation to ensure public safety and adherence to regulatory standards. This analysis suggests that while bottled water presents a lower risk for PFAS exposure, tap water may pose a higher concern, necessitating concerted efforts to mitigate contamination sources within municipal water systems.

### 3.2. Non-Regulated PFASs

**Figure 2:**
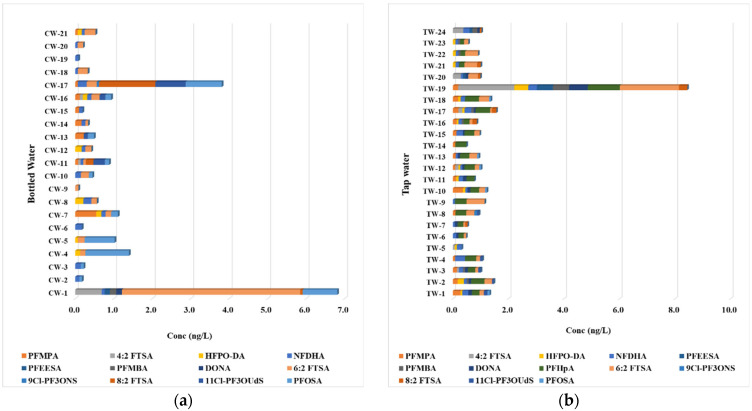
Unregulated PFAS levels determined in (**a**) bottled water and (**b**) tap water.

The analysis of unregulated per- and polyfluoroalkyl substances (PFASs) in bottled water, as presented in the data, sheds light on the complex environmental and public health issues surrounding these compounds. The results from multiple bottled water samples demonstrate the presence of various PFASs, including 4:2 Fluorotelomer Sulfonic Acid (4:2 FTSA), Hexafluoropropylene Oxide Dimer Acid (HFPO-DA), and other compounds, highlighting the widespread contamination that is not yet addressed by current EU regulations (Figure 2a).

The detection of 4:2 FTSA in several samples, with a maximum concentration of 0.689 ng/L observed in CW-1, raises important concerns regarding the potential for these compounds to accumulate over time, especially as they are not subject to the stringent regulatory controls applied to longer-chain PFASs. While individual concentrations are below the regulatory limits set by the EU Drinking Water Directive (DWD), the cumulative effects of exposure to multiple unregulated PFAS compounds remain largely unassessed and may pose significant health risks (Table S8). HFPO-DA similarly illustrates a pattern of contamination, with detectable levels observed in samples CW-4 and CW-9, albeit at low concentrations. This demonstrates that while current regulations focus on specific, well-characterized compounds, a myriad of unregulated PFASs—like HFPO-DA—continue to enter the water supply, likely through various pathways including industrial runoff and environmental degradation of more persistent precursors. The presence of 9Cl-PF3ONS, a relatively newly identified sulfonamide compound, further exemplifies the challenges faced in monitoring and regulating emerging PFASs. With higher concentration peaks (4.64 ng/L in CW-1) compared to other compounds, this raises critical questions about the potential endocrine-disrupting effects of such substances, especially given their environmental persistence and propensity for bioaccumulation. Notably, the frequencies of detection for these compounds across different bottled water brands emphasize the necessity for ongoing surveillance and research. Bottled water, which is often perceived as a safer alternative to tap water, can still harbor significant PFAS concentrations that are currently undefined by regulatory frameworks. This underscores a critical gap in public health protection, as consumers may unwittingly expose themselves to harmful substances without any regulatory oversight.

The comprehensive analysis of the presence of unregulated per- and polyfluoroalkyl substances (PFASs) in tap water samples highlights the complexities surrounding these emerging contaminants that currently fall outside the scope of European Union regulation (Table S9, Figure 2b). The data obtained from 24 tap water samples reveal detectable concentrations of various PFAS, including PFMPA, 4:2 FTSA, HFPO-DA, NFDHA, PFEESA, PFMBA, and others, indicating a persistent prevalence of these substances in municipal water supplies.

The concentrations of PFAS compounds varied across the samples, with notable findings such as 4:2 FTSA, which was present in several samples, including a striking concentration of 2.02 ng/L detected in TW-19. This level raises significant concerns, especially since this compound is a precursor to long-chain PFASs. Its presence may indicate broader contamination trends exacerbated by inadequate wastewater management practices and industrial discharges. Additionally, HFPO-DA was detected in multiple samples, showcasing a widespread occurrence that aligns with recent research indicating an increase in the environmental presence of this emerging contaminant. Interestingly, PFMPA was consistently detected at low levels, with concentrations ranging from 0.08 to 0.28 ng/L across several samples. While these levels are below regulatory limits, they underscore a growing necessity for assessing cumulative exposure risks associated with long-term tap water consumption containing trace levels of multiple unregulated PFASs. Given the complex nature of PFAS chemistry and their potential synergistic effects, it is imperative to recognize that even low-level exposure to a mixture of these substances may pose risks that are not yet fully understood, particularly concerning human health. Among the notable samples, TW-19 displayed a diverse profile of PFAS contamination, with multiple compounds detected, including NFDHA (0.31 ng/L) and PFEESA (0.58 ng/L), in addition to the high concentration of 4:2 FTSA. This robust presence of various PFASs highlights the challenge that municipalities face in delivering safe drinking water, as these substances can enter the water supply through different pathways, including runoff from landfills, industrial sites, and even atmospheric deposition.

The comparative analysis of unregulated per- and polyfluoroalkyl substances (PFASs) in bottled versus tap water, as summarized in Table 2, reveals pertinent insights into the prevalence and concentration of these contaminants in both water sources. This discussion underscores the implications of these findings regarding public health and regulatory oversight. Bottled water demonstrated varying concentrations of unregulated PFASs, with the sum of detections (Σ unregulated PFASs) peaking at 10.20 ng/L and an average concentration of 3.00 ng/L across samples. Notably, the median concentration was 1.67 ng/L, suggesting that while a significant portion of bottled water contained some level of contamination, the overall exposure might not be uniformly distributed. In comparison, the highest concentrations of individual PFASs, particularly 6:2 FTSA, reached up to 4.64 ng/L in bottled water, indicating a notable presence of this substance, which has garnered increasing attention due to its implications for human health. Conversely, tap water samples exhibited a different profile. The highest overall detection of Σ unregulated PFASs was lower than that of bottled water, with a maximum concentration of 7.78 ng/L and a mean concentration of 2.25 ng/L. Despite the lower maximum concentration, a greater detection frequency was noted for several PFAS compounds in tap water. For instance, NFDHA showed a frequency of 92%, indicating that this compound is nearly ubiquitous in the monitored tap water samples, reflecting potential systemic contamination issues affecting broader water supply systems. Notably, NFDHA was not detected in the blank samples, confirming that its presence in tap water is not due to background contamination introduced during sampling or analysis.

Analyzing specific PFAS compounds reveals that PFMPA was detected in 38% of bottled water samples, with a maximum concentration of 0.55 ng/L and a much higher frequency of 63% in tap water at concentrations reaching 0.36 ng/L. This discrepancy raises questions about the sources of these contaminants, suggesting that tap water may be more prone to routine exposure to PFASs from environmental sources or industrial activities. In contrast, bottled water may involve different sourcing practices that could influence contamination levels. Furthermore, the observed presence of 4:2 FTSA underscores the significance of monitoring this contaminant, as it was found in 14% of bottled water samples, with a maximum value of 0.69 ng/L. In contrast, in tap water, it was substantially more prevalent at a maximum of 2.02 ng/L with a frequency of 29%. This finding is alarming given the potential implications of long-term exposure to such substances, particularly as 4:2 FTSA is a precursor compound with the potential to degrade into more harmful forms. Compounds such as PFEESA and PFMBA were also detected in tap water at higher maximum concentrations (0.58 ng/L) compared to their bottled water counterparts. This trend might suggest inefficiencies in purification processes for tap water or variable contamination across different sources and distribution systems.

The findings highlight a complex landscape of unregulated PFAS contamination across bottled and tap water. While bottled water is often perceived as a safer alternative, detectable levels of various unregulated PFASs reveal that this assumption may not be entirely warranted. The data indicate that, while bottled water has specific constituents that can impact its safety profile, tap water poses significant risks due to the broad range of unregulated PFASs also present with high detection frequencies. This necessitates the ongoing need for regulatory authorities to reassess existing frameworks to encapsulate various unregulated PFASs, enhancing monitoring protocols and public health protections for both bottled and tap water sources. Through more comprehensive data collection and safety assessments, stakeholders can better inform consumers and promote safer water consumption practices.

### 3.3. Co-Occurrence of PFASs

**Figure 3:**
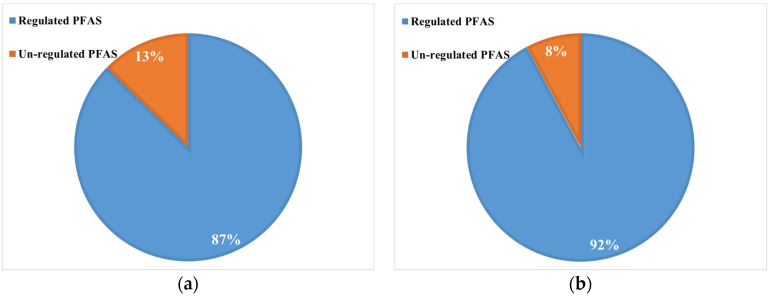
Distribution between regulated and unregulated PFASs determined in (**a**) bottled water and (**b**) tap water.

The analysis of PFAS contamination in bottled and tap water reveals a notable distinction in the distribution of regulated and unregulated compounds, with regulated PFASs constituting 87% of the total PFAS concentration in bottled water and 92% in tap water. This suggests that regulatory frameworks should primarily target well-known PFAS compounds, ensuring their monitoring in both water sources. However, the presence of unregulated PFASs, accounting for 13% in bottled water and 8% in tap water, highlights an ongoing gap in oversight, as these emerging contaminants continue to infiltrate drinking water systems (Figure 3a,b). The higher occurrence of unregulated PFASs in bottled water suggests potential contamination pathways linked to diverse water sourcing, including groundwater, surface water, or the bottling process itself. In contrast, tap water’s elevated concentration of regulated PFASs may indicate the effectiveness of municipal treatment processes in addressing these compounds while still leaving room for the presence of unregulated PFASs, potentially originating from legacy pollution or ongoing industrial discharges. The co-occurrence of both regulated and unregulated PFASs raises critical concerns regarding cumulative exposure and potential synergistic effects, given that many of these substances have not been fully evaluated for their toxicological impact.

PFASs are known for their environmental persistence and bioaccumulative properties, with several compounds such as PFOA and PFOS linked to adverse health outcomes including liver toxicity, immunotoxicity, developmental effects, and increased risk of cancer [31,32]. Emerging PFASs, including novel replacements like GenX and ADONA, are increasingly detected in water systems, yet toxicological data remain limited. Initial studies have suggested that these compounds may exhibit similar or even greater toxicity compared to legacy PFASs, including potential endocrine disruption and hepatotoxicity [33,34]. Regulatory actions are evolving, with the U.S. EPA recently establishing Maximum Contaminant Levels (MCLs) for six PFASs, including PFOA and PFOS, under the National Primary Drinking Water Regulation. However, thousands of PFASs remain unregulated globally, prompting calls for class-based regulation approaches to address cumulative exposure risks [24,35]. Given their long biological half-lives and potential for mixture toxicity, chronic low-level exposure to both regulated and unregulated PFASs may pose underestimated health risks, particularly for vulnerable populations such as infants and pregnant women [36].

While regulatory efforts focus on known PFASs, the presence of unregulated compounds introduces uncertainty regarding long-term health risks, particularly when individuals are exposed to a mixture of PFASs in drinking water. The patterns observed suggest that while regulatory frameworks play a role in mitigating contamination, they may not comprehensively address the full spectrum of PFAS chemicals, emphasizing the need for expanded monitoring and stricter regulations to ensure public health protection.

**Figure 4:**
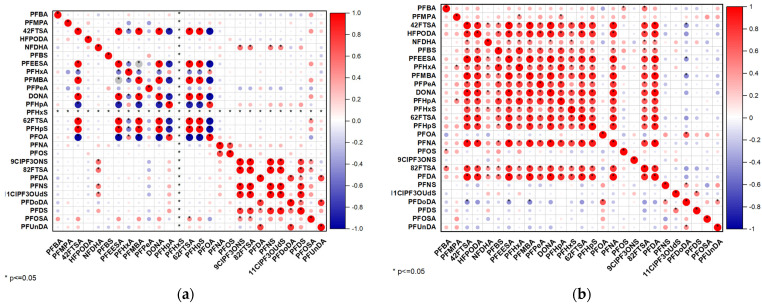
Pearson correlation (**a**) bottled water and (**b**) tap water.

The Pearson correlation matrices for PFAS compounds in bottled water (Figure 4a) and tap water (Figure 4b) provide valuable insights into the co-occurrence patterns and relationships between these persistent pollutants in different water sources. In both matrices, the correlations are visually represented using a color gradient, where red indicates positive correlations and blue represents negative correlations. The circles’ size and intensity correspond to the correlation’s strength, with larger and darker circles signifying stronger associations between PFAS compounds.

A key observation from both figures is the predominance of strong positive correlations among several PFAS compounds, as evidenced by the widespread red circles across the matrices. This suggests that many PFAS species co-occur in bottled and tap water, likely due to shared contamination sources, typical industrial applications, or similar physicochemical properties that govern their environmental behavior. The consistent clustering of positively correlated compounds implies that certain groups of PFASs originate from similar manufacturing processes or contamination events, leading to their concurrent presence in drinking water sources. While the general trend of strong positive correlations is observed in bottled and tap water, some differences emerge in the clustering patterns. Bottled water has a slightly greater correlation strength variation, with some PFAS compounds showing weaker or even negative correlations. This could be attributed to differences in treatment processes, source variations, or bottle material interactions that may influence PFAS leaching. Conversely, tap water exhibits a more uniform pattern of strong positive correlations, particularly in the upper left section of the matrix, where a distinct subset of PFAS compounds consistently appears together. This could be linked to municipal water sources affected by industrial discharges, legacy contamination, or degradation products of precursor molecules. Another notable difference is the presence of negative correlations, which are more evident in bottled water compared to tap water, as indicated by the presence of blue-colored circles. These negative correlations, although relatively weak, may suggest variations in degradation pathways, competitive adsorption and desorption behaviors, or different source contributions. In contrast, tap water samples exhibit very few negative correlations, implying that competitive interactions among PFAS compounds are minimal in these water systems, potentially due to their stability and persistence in aqueous environments.

The statistical significance of the observed correlations further supports the reliability of these findings. The presence of black dots indicates statistically significant correlations (*p* < 0.05), emphasizing that the co-occurrence of PFAS compounds is unlikely to be random. This statistical robustness strengthens the interpretation that certain PFAS compounds are linked through environmental or industrial pathways, with distinct patterns observed for short-chain and long-chain PFASs. These patterns align with known differences in their persistence, mobility, and bioaccumulation potential, which may contribute to variations in their correlation structures between bottled and tap water [11,37].

Overall, the combined analysis of PFAS correlations in bottled and tap water highlights these compounds’ widespread presence and persistence in drinking water systems. The strong positive correlations suggest that PFAS contamination is often multifaceted, involving multiple compounds rather than isolated occurrences. The slight differences in correlation patterns between bottled and tap water underscore the influence of factors such as source contamination, treatment methods, and environmental transport mechanisms. These findings emphasize the need for comprehensive monitoring strategies and regulatory measures to assess and mitigate PFAS contamination in drinking water, ensuring public health protection against these persistent environmental pollutants [11].

Principal Component Analysis (PCA) assessed the variability and distribution of per- and polyfluoroalkyl substances (PFASs) in bottled and tap water samples. The PCA score plots provide a visualization of sample clustering based on PFAS composition, while the loading plots elucidate the contribution of individual PFAS compounds to the observed variance. This multivariate approach facilitates the identification of contamination patterns, potential sources, and differences in PFAS distribution across water sources.

**Figure 5:**
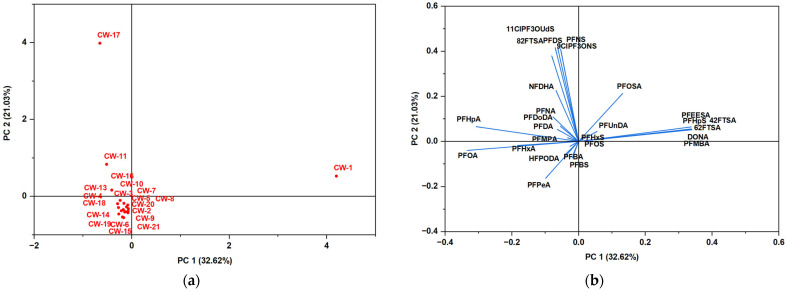
PCA assessed on PFAS results in bottled water: (**a**) PCA score plot for bottled water samples; (**b**) PCA loading plot showing the contribution of individual PFAS compounds to the total variance.

Figure 5a presents the PCA score plot for bottled water samples, where the first principal component (PC1) and the second principal component (PC2) account for 32.62% and 21.03% of the total variance, respectively. The majority of bottled water samples cluster near the origin, suggesting a relatively homogeneous PFAS composition among these brands. However, certain samples (CW-1, CW-11, and CW-17) deviate from the main cluster, indicating significant differences in PFAS contamination profiles. These deviations may be attributed to variations in water sources, treatment methodologies, or contamination pathways, which could be influenced by differences in bottling practices or environmental factors.

Figure 5b, the PCA loading plot, depicts the contributions of individual PFAS compounds to the observed variance. Specific compounds, including PFOSA, PFUnDA, and PFBS, exhibit strong positive loadings along PC1 and PC2, suggesting their elevated concentrations in samples positioned in these quadrants. In contrast, PFAS compounds such as PFOA and PFPeA are located near the origin, implying their relatively uniform distribution across most bottled water samples. The alignment of certain PFAS compounds in similar directions suggests co-occurrence patterns, potentially indicative of shared contamination sources or transport mechanisms. These findings demonstrate that while most bottled water samples exhibit comparable PFAS profiles, a subset displays distinct characteristics, likely due to differences in production processes or environmental influences.

**Figure 6:**
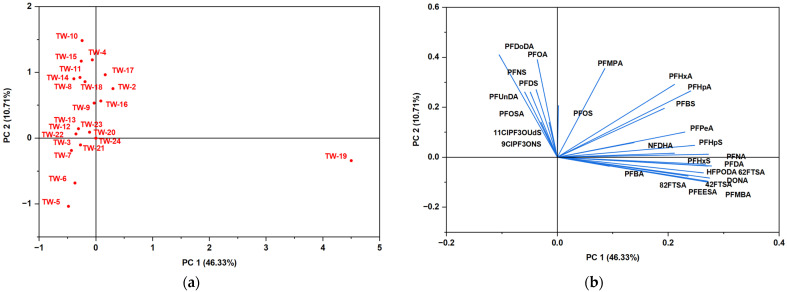
PCA assessed on PFAS results in tap water: (**a**) PCA score plot of tap water samples showing overall similarity with a few outliers. (**b**) Loading plot highlighting key PFAS compounds contributing to variance, including PFOS and PFOA.

Figure 6a presents the PCA score plot for tap water samples, with PC1 and PC2 explaining 46.33% and 10.71% of the total variance, respectively. Like bottled water, most tap water samples are clustered near the origin, signifying comparable PFAS contamination profiles across different municipal water supplies. However, a few outlier samples, such as TW-19, are positioned further from the central cluster, indicating substantial differences in PFAS composition. These variations may stem from differences in contamination sources, water treatment technologies, or regional disparities in PFAS exposure levels. The PCA loading plot in Figure 6b provides insights into the contributions of individual PFAS compounds to the variance observed in the dataset. Several PFAS species, including PFOS, PFOA, and long-chain perfluoroalkyl acids, exhibit strong loadings along PC1 and PC2, suggesting their significant role in differentiating tap water samples. The clustering of specific PFAS compounds along particular vectors implies potential correlations, likely arising from common contamination pathways, such as industrial discharge, legacy pollution, or variations in regulatory standards and treatment methodologies.

The PCA results indicate that bottled and tap water samples generally exhibit similar PFAS contamination profiles, with a subset of samples displaying distinct compositions. Identifying outliers and key PFAS contributors highlights the complexity of PFAS contamination and underscores the need for continuous monitoring and regulatory oversight to ensure drinking water safety. Further investigations into the sources and transport mechanisms of PFASs in water systems are warranted to develop effective mitigation strategies.

### 3.4. Comparison of PFAS Values Determined in Tap and Bottled Water Around the World

PFAS have been detected worldwide in tap and bottled water, with varying concentrations depending on the geographical region and the specific compounds analyzed. The data presented highlight significant differences in PFAS contamination between tap and bottled water sources across different countries.

The concentration of PFAS in tap water varies widely, with some of the highest values reported in Spain [38] at 258 ng/L for PFOS, the Czech Republic [39] at 108 ng/L for PFOA, and France [40] at 32.9 ng/L for PFHpA. The lowest concentrations were observed in Turkey [41] with 0.1–2.9 ng/L and Sweden [42] with 0.1–4.6 ng/L. The current study reports a maximum PFAS concentration of 17.2 ng/L, with PFOA as the dominant compound. In comparison, PFAS concentrations in bottled water tend to be lower. One of the highest previously reported values was in China [43], where PFOA was detected at 1.50 ng/L. The current study reported even higher concentrations, with 4.48 ng/L for PFOA and 5.15 ng/L for PFHpS. Other studies, such as those in France [44] and Norway [45,46], have reported significantly lower concentrations, at <LOD–0.28 ng/L and <LOD–0.42 ng/L, respectively.

Generally, PFAS levels in tap water are higher than in bottled water. The highest recorded values in tap water, such as in Spain, the Czech Republic, and France, are significantly greater than those in bottled water. PFOA and PFOS are among the most frequently detected PFAS compounds. In tap water, PFOA was particularly prominent in the Czech Republic [39] at 108 ng/L and in France [40] at 17.6 ng/L, while PFOS was dominant in Spain [38] at 258 ng/L. PFOA was the dominant compound in multiple studies in bottled water, with the highest concentration in the USA at 13 ng/L [17]. European countries show a wide range of PFAS contamination, with some regions like Spain and the Czech Republic exhibiting notably high concentrations. In contrast, other areas, such as the UK [47,48] and Sweden [42], have much lower levels. In bottled water, the variations are less pronounced, with generally lower concentrations across all reported studies. The current study’s findings (<LOQ–17.2 ng/L for tap water and <LOQ–4.48 ng/L for bottled water) align with global trends, confirming that tap water generally contains higher PFAS levels than bottled water. The dominant presence of PFOA in both water sources further supports previous research on the widespread prevalence of this compound. Overall, while bottled water appears to contain lower PFAS concentrations than tap water, the persistence of these contaminants in both sources underscores the necessity for stringent water quality monitoring and mitigation strategies worldwide.

### 3.5. Human Exposure to PFAS via Drinking Water

A human health risk assessment evaluated potential risks associated with PFAS exposure through drinking water consumption. This assessment estimates the estimated daily intake (EDI) of PFASs and compares it with established tolerable daily intake (TDI) levels to determine potential health risks. The EDI of PFASs, expressed in ng/kg body weight/day, was calculated using Equation (1), incorporating PFAS concentrations in water (Cw), daily water consumption (Dw), and body weight (Bw). This formula is widely used in risk assessments for environmental contaminants, ensuring consistency with established protocols (e.g., U.S. EPA guidelines). Drinking water consumption rates and average body weights for different age groups and sexes, as detailed in Table S10, were used to refine the EDI calculations, ensuring a more accurate estimation of exposure across different population groups.

The estimated daily intake (EDI) values for various per- and polyfluoroalkyl substances (PFASs) were calculated for different age groups and sexes based on their respective drinking water consumption and body weight. The results highlight significant differences in PFAS exposure between bottled and tap water sources and variations across demographic groups (Table S11). The calculation of EDI incorporated age-specific water consumption rates and average body weight values (as detailed in Table S10). This allowed for more accurate comparisons of exposure levels across different population groups. In bottled water, the EDI values for most PFAS compounds remain relatively low across all age groups, with minor variations between males and females [49]. Among the analyzed compounds, perfluorooctanoic acid (PFOA) exhibits the highest EDI values, ranging from 74.3 ng/kg bw/day in teenage males to 121 ng/kg bw/day in children. Similarly, perfluorooctane sulfonamide (PFOSA) and perfluorobutanesulfonic acid (PFBS) also show elevated EDI values compared to other compounds, particularly in children. The lower EDI values observed in bottled water suggest that contamination levels remain relatively consistent across different age groups, with slight fluctuations due to differences in body weight and water intake.

Conversely, tap water demonstrates higher EDI values for nearly all PFAS compounds, with children exhibiting the highest exposure levels due to greater water consumption relative to body weight. PFOA remains the most concerning contaminant, with EDI values reaching 464 ng/kg bw/day in children, followed by perfluorodecane sulfonic acid (PFDS) at 238 ng/kg bw/day and perfluorododecanoic acid (PFDoDA) at 194 ng/kg bw/day. The pronounced differences between bottled and tap water suggest a higher degree of PFAS contamination in municipal water sources, which environmental factors, industrial discharges, or inadequate treatment processes may influence. Age-dependent variations in PFAS exposure are also evident in bottled and tap water. Children consistently exhibit the highest EDI values due to their lower body weight and higher per-unit water consumption, while adults and seniors display comparatively lower values. These findings align with previous studies indicating that younger populations are more vulnerable to PFAS exposure due to increased ingestion rates relative to body weight [50,51].

The evaluation of human health risks associated with PFAS exposure in drinking water was performed using the Risk Index (RI), which compares EDI values to tolerable daily intake (TDI) thresholds (Table 3). The RI provides a relative measure of risk, where values greater than 1.0 indicate potential health risks. The results reveal significant variations in risk levels across different PFAS compounds, age groups, and water sources. Among the analyzed PFAS, PFOA presents the highest RI values in bottled and tap water, with children exhibiting the most significant risk (RI ≈ 192). This finding is concerning, as the calculated RI for PFOA substantially exceeds unity, suggesting potential for adverse health effects, particularly among younger populations. Perfluorooctane sulfonic acid (PFOS) and perfluorononanoic acid (PFNA) also exhibit elevated RI values, reaching 4.05 and 4.74 in children, respectively, indicating potential health risks due to chronic exposure. The observed trend across all PFAS compounds demonstrates that younger age groups consistently exhibit higher RI values due to their greater relative water consumption per unit of body weight [49,50].

A comparison between bottled and tap water sources shows that RI values remain nearly identical, indicating that the PFAS concentrations in bottled and tap water contribute similarly to overall exposure. This suggests that contamination in bottled water is not significantly lower than in municipal supplies, raising concerns about the effectiveness of purification processes in commercial drinking water products. Product labels indicate that most bottled water samples originated from natural springs. In contrast, tap water was supplied by municipal systems sourcing water from a mix of groundwater, springs, or treated surface waters. The variability in PFAS levels observed across tap water samples may reflect differences in local pollution sources, regional industrial activity, and variation in treatment efficiency. However, due to the lack of publicly available and standardized information on specific treatment technologies used by either bottled water producers or municipal utilities, it is not easy to draw definitive conclusions regarding the role of treatment in PFAS reduction. This limitation highlights the need for greater transparency in water treatment reporting and regulatory oversight of PFAS contamination across all drinking water sources.

While most of the short-chain PFAS compounds, such as PFBA and PFHxA, exhibit RI values well below unity, indicating a lower risk, the presence of compounds such as PFDoDA and PFHxS with RI values nearing 0.6 in some groups suggests the need for closer regulatory scrutiny. The relatively high RI values observed for PFOSA and PFDS further highlight the potential health implications of long-chain PFASs, which tend to accumulate in the body. The results of this risk assessment emphasize the need for stricter regulations on PFAS contamination in drinking water. The consistently high RI values for certain PFASs, particularly PFOA, PFOS, and PFNA, raise significant public health concerns, especially for vulnerable populations such as children, who are more susceptible to the adverse effects of these chemicals due to their developing bodies and higher water consumption relative to body weight. Differences in risk between genders and age groups further highlight the need for targeted interventions. For instance, while children may face greater risks due to their increased exposure, women of reproductive age might also be more vulnerable to certain PFASs, which can affect hormonal balance and fetal development. These findings underscore the importance of improved water treatment technologies, routine monitoring of PFAS levels in both bottled and tap water, and establishing more stringent limits to mitigate long-term exposure risks, particularly for the most vulnerable segments of the population.

While this study provides important insights into PFAS exposure levels through drinking water, it is essential to recognize the inherent uncertainties associated with Estimated Daily Intake (EDI) and Tolerable Daily Intake (TDI) estimates. Multiple factors influence these estimates, including variability in water consumption across different populations, body weight differences, and the accuracy of PFAS concentration measurements. The lack of standardized and region-specific data on drinking water consumption, particularly for distinct age and gender groups, adds another layer of uncertainty. Furthermore, variations in the analytical techniques used to detect and quantify PFAS compounds across laboratories may lead to differences in reported concentrations, further contributing to the uncertainty of exposure estimates. Therefore, it is crucial to interpret the risk assessments cautiously, recognizing that the estimates here represent the best available data but may be subject to future refinement as more accurate information becomes available.

Another key consideration is the potential for additive or synergistic effects of multiple PFAS exposures. This study primarily evaluates individual PFAS compounds, but humans are likely exposed to a mixture of different PFASs over time. The additive or even synergistic effects of these compounds may amplify the health risks associated with their exposure. For example, different PFASs may share common toxicological pathways, leading to greater adverse effects when they occur together in the environment. This aspect of PFAS contamination remains an under-researched area, and future studies should explore the combined impact of PFAS mixtures to more accurately assess the potential health risks of exposure to multiple compounds simultaneously [51,52].

Lastly, this risk assessment is focused on PFAS exposure from drinking water alone. However, cumulative risk assessment, which considers other sources of PFAS exposure such as food, air, and consumer products, is crucial for understanding the total burden of PFASs on human health. Individuals are exposed to PFASs from multiple sources, and drinking water represents only one of these potential exposures. A comprehensive evaluation of cumulative exposure would provide a more complete picture of the overall health risks associated with PFASs and help inform more effective public health policies and regulations. It is recommended that future research adopt a broader approach to risk assessment, incorporating multiple exposure pathways to better inform regulatory standards and public health recommendations [53,54,55].

## 4. Strengths and Limitations

The strengths and limitations of the study regarding the analysis of per- and polyfluoroalkyl substances (PFASs) in bottled and tap water provide a nuanced understanding of the findings and their implications. One of the significant strengths of this study lies in its comprehensive sampling strategy, which included a diverse array of bottled water brands and municipal tap water sources. By analyzing 21 bottled and 24 tap water samples, the study captures a broad spectrum of potential PFAS contamination, enhancing the generalizability of the findings. Focusing on regulated and unregulated PFAS compounds provides a more holistic view of contamination levels. It underscores the issue’s complexity, particularly concerning emerging contaminants that often escape regulatory scrutiny. Rigorous analytical methods to quantify PFAS concentrations ensure that the data gathered are reliable and facilitate meaningful comparisons between the two water types. In addition, the study benefits from a systematic risk assessment approach that estimates daily intake levels for various PFAS across different demographic groups. This analysis strengthens the link between environmental exposure and potential public health implications, particularly for vulnerable populations such as children. The study effectively highlights the health risks associated with PFAS exposure through drinking water by utilizing a robust framework to calculate estimated daily intakes and risk indices.

However, the study also has limitations that should be acknowledged. One notable limitation is the small sample size, particularly for bottled water, which may not encompass the full diversity of bottled water sources available on the market. This limitation restricts the ability to draw broad conclusions on the prevalence of PFAS contamination in bottled water. Additionally, the study’s focus on specific geographic regions may limit the applicability of the findings to other areas, where different environmental and regulatory factors could influence PFAS concentrations. Another limitation is the potential for variability in water treatment practices and source water quality, which were not explicitly controlled in the study. Such variability may contribute to differences in PFAS levels that are not fully accounted for, thereby complicating the interpretation of the results. Furthermore, while the study assesses a range of known PFAS compounds, the evolving landscape of emerging PFASs necessitates ongoing research to identify and quantify new compounds and their potential health implications. Lastly, the assessment of human exposure, while informative, relies on estimates based on average consumption patterns that may not accurately reflect individual behaviors. Variability in dietary habits, local water quality, and individual demographic factors could result in a more complex exposure scenario that this study may not fully capture.

In addition to these points, several potential confounding factors should be considered. One such factor is the packaging material of bottled water, particularly the type of plastic used, which may influence PFAS migration into water during storage [56]. Seasonal variability and weather conditions at the time of sampling could also affect PFAS levels, especially in surface water-derived sources, due to changes in runoff and dilution [57]. Furthermore, the presence of co-contaminants such as organic matter or heavy metals in water may interfere with PFAS quantification or affect their bioavailability and behavior in treatment processes [58]. Another confounding aspect relates to the analytical detection limits and potential matrix effects that might lead to underestimation or overestimation of certain PFASs, particularly at low concentrations. While quality control protocols were applied, such analytical uncertainties remain a technical constraint inherent in PFAS monitoring studies [59]. Lastly, differences in the manufacturing processes and water source declarations by bottling companies, which were not publicly disclosed in all cases, limit the ability to trace contamination pathways with high certainty [60].

The strengths of this study are significant in providing valuable insights into PFAS contamination in drinking water, while the limitations highlight the need for continued research and monitoring in this critical area of environmental health.

## 5. Conclusions

The comprehensive analysis of per- and polyfluoroalkyl substances (PFASs) in bottled and tap water reveals significant insights into the prevalence, contamination levels, and public health implications associated with these emerging environmental pollutants. Although bottled water is often perceived as a safer alternative, the findings demonstrate that both bottled and tap water contain detectable levels of regulated and unregulated PFASs, underscoring the complexity of PFAS contamination in drinking water sources.

The results indicate that regulated PFAS compounds comprise the majority of detected substances in both bottled (87%) and tap water (92%). This highlights the effectiveness of current regulatory frameworks in addressing well-characterized contaminants. However, the presence of unregulated PFASs—13% in bottled water and 8% in tap water—reveals a notable gap in oversight, suggesting that these emerging contaminants continue to infiltrate drinking water systems with potential health risks that are not yet fully understood. Specifically, the analysis highlights PFOA as a predominant contaminant, with alarmingly high detection levels in both water types, particularly in tap water, which exhibited a maximum concentration of 17.163 ng/L. This level is of concern given the known health risks associated with PFOA, including endocrine disruption and developmental issues. Moreover, significant variances in PFAS concentrations and detection frequencies between bottled and tap water suggest the influence of environmental sources, treatment processes, and local regulations on water quality. The presence of short-chain PFASs, including PFBA and PFBS, was notably higher in tap water, indicating localized contamination sources, potentially linked to industrial discharges. In contrast, bottled water exhibited variability in PFAS profiles, influenced by sourcing and bottling practices. These findings have critical implications for water safety policy and consumer awareness. The continued detection of unregulated PFASs suggests the need for proactive regulatory action before long-term health consequences become more widespread. The study highlights the importance of addressing not only legacy PFASs but also emerging compounds, many of which remain unmonitored and unregulated. Cumulative exposure to PFASs, given their potential synergistic effects, necessitates urgent attention from regulatory authorities, urging a reassessment of existing monitoring frameworks to address emerging contaminants.

The human health risk assessment underscored the urgency of this matter, revealing that children are particularly vulnerable, exhibiting the highest estimated daily intake values for various PFAS compounds. The risk indices calculated for PFOA and other long-chain PFASs in both water sources indicate significant potential for adverse health effects, especially in younger populations.

Given these outcomes, the study serves as a scientific basis for strengthening drinking water standards, informing public health risk communication, and prioritizing investments in advanced water treatment technologies. The evidence presented supports the inclusion of more PFAS compounds in routine monitoring programs and emphasizes the need for harmonized international regulations. Overall, this analysis highlights the critical need for ongoing surveillance and regulation of PFASs in drinking water. The evidence calls for more stringent limits on PFAS concentrations, improvements in water treatment technologies, and comprehensive monitoring efforts to protect public health from the harmful effects of these persistent pollutants. As the understanding of PFAS toxicity and exposure risks evolves, regulatory measures must evolve in tandem, ensuring that both bottled and tap water remain safe for consumption. In summary, while bottled water may offer some advantages, the overall risk of PFAS exposure through both sources underscores the need for greater vigilance and effective management strategies to safeguard public health.

## Figures and Tables

**Table 1:** Summary statistics for regulated PFAS concentrations in bottled and tap water samples. Reported values include minimum (Min), maximum (Max), mean, and median concentrations (all in ng/L), along with detection frequency (Freq, %).

PFAS	Bottled Water					Tap Water				
	Min	Max	Mean	Median	Freq	Min	Max	Mean	Median	Freq
PFBA	<LOQ	0.34	0.15	0.09	33	<LOQ	1.96	0.43	0.21	75
PFBS	<LOQ	0.70	0.70	0.70	5	<LOQ	1.62	0.69	0.80	46
PFHxA	<LOQ	0.29	0.24	0.25	86	<LOQ	0.88	0.31	0.27	83
PFPeA	<LOQ	0.42	0.22	0.21	86	<LOQ	1.08	0.29	0.26	79
PFHpA	<LOQ	0.26	0.22	0.21	95	<LOQ	1.16	0.37	0.34	88
PFHxS	<LOQ	<LOQ	<LOQ	<LOQ	0	<LOQ	<LOQ	<LOQ	<LOQ	17
PFHpS	<LOQ	4.15	0.46	0.18	67	<LOQ	0.93	0.26	0.25	79
PFOA	<LOQ	4.48	4.04	4.03	95	1.88	17.16	8.95	8.57	100
PFNA	<LOQ	0.11	0.08	0.07	38	<LOQ	0.85	0.15	0.11	96
PFOS	<LOQ	0.09	0.09	0.09	5	<LOQ	0.45	0.21	0.20	46
PFDA	<LOQ	0.10	0.07	0.07	24	<LOQ	1.69	0.31	0.08	75
PFNS	<LOQ	0.39	0.19	0.13	14	<LOQ	0.09	0.08	0.08	38
PFDoDA	<LOQ	0.51	0.18	0.13	38	<LOQ	0.28	0.20	0.21	83
PFDS	<LOQ	0.35	0.19	0.12	24	<LOQ	0.12	0.09	0.08	42
PFUnDA	<LOQ	0.32	0.12	0.10	76	<LOQ	0.18	0.11	0.10	75
ΣPFAS	4.48	6.53	5.34	5.17	-	2.57	19.2	10.8	10.4	-

**Table 2:** Summary statistics for unregulated PFAS concentrations in bottled and tap water samples. Reported values include minimum (Min), maximum (Max), mean, and median concentrations (all in ng/L), along with detection frequency (Freq, %).

Unregulated PFAS	Bottled Water					Tap Water				
	Min	Max	Mean	Median	Freq	Min	Max	Mean	Median	Freq
PFMPA	0.06	0.55	0.17	0.11	38	<LOQ	0.36	0.15	0.12	63
4:2 FTSA	0.06	0.69	0.28	0.08	14	<LOQ	2.02	0.50	0.22	29
HFPO-DA	0.07	0.21	0.13	0.13	33	<LOQ	0.50	0.12	0.09	54
NFDHA	0.06	0.23	0.11	0.10	81	<LOQ	0.36	0.15	0.12	92
PFEESA	<LOQ	0.14	0.14	0.14	5	<LOQ	0.58	0.26	0.10	13
PFMBA	<LOQ	0.17	0.17	0.17	5	<LOQ	0.58	0.18	0.09	33
DONA	<LOQ	0.15	0.15	0.15	5	<LOQ	0.67	0.14	0.09	58
6:2 FTSA	<LOQ	4.64	0.47	0.16	71	<LOQ	2.12	0.36	0.21	79
9Cl-PF3ONS	<LOQ	0.06	0.06	0.06	5	<LOQ	0.10	0.10	0.10	4
8:2 FTSA	<LOQ	1.47	0.58	0.20	14	<LOQ	0.30	0.14	0.13	71
11Cl-PF3OUdS	<LOQ	0.79	0.33	0.22	19	<LOQ	0.09	0.08	0.08	21
PFOSA	<LOQ	1.13	0.42	0.16	46	<LOQ	0.08	0.07	0.06	21
Σ unregulated PFASs	0.25	10.20	3.00	1.67	-	<LOQ	7.78	2.25	1.40	-

**Table 3:** Risk Index (RI) values for PFAS exposure by age group and sex, based on estimated daily intake (EDI) from bottled and tap water consumption.

PFAS	RI Bottled Water								RI Tap Water							
	Kids (6–11 Years)		Teenagers (12–19 Years)		Adults (20–60 Years)		Seniors (>60 Years)		Kids (6–11 Years)		Teenagers (12–19 Years)		Adults (20–60 Years)		Seniors (>60 Years)	
	M	F	M	F	M	F	M	F	M	F	M	F	M	F	M	F
PFBA	6.1 × 10^−4^	6.1 × 10^−4^	3.7 × 10^−4^	3.4 × 10^−4^	4.0 × 10^−4^	4.6 × 10^−4^	3.8 × 10^−4^	4.1 × 10^−4^	6.1 × 10^−4^	6.1 × 10^−4^	3.7 × 10^−4^	3.4 × 10^−4^	4.0 × 10^−4^	4.6 × 10^−4^	3.8 × 10^−4^	4.1 × 10^−4^
PFHxA	7.9 × 10^−5^	7.9 × 10^−5^	4.9 × 10^−5^	4.4 × 10^−5^	5.2 × 10^−5^	6.0 × 10^−5^	5.0 × 10^−5^	5.3 × 10^−5^	7.9 × 10^−5^	7.9 × 10^−5^	4.9 × 10^−5^	4.4 × 10^−5^	5.2 × 10^−5^	6.0 × 10^−5^	5.0 × 10^−5^	5.3 × 10^−5^
PFHpA	6.9 × 10^−5^	6.9 × 10^−5^	4.2 × 10^−5^	3.9 × 10^−5^	4.5 × 10^−5^	5.3 × 10^−5^	4.4 × 10^−5^	4.6 × 10^−5^	6.9 × 10^−5^	6.9 × 10^−5^	4.2 × 10^−5^	3.9 × 10^−5^	4.5 × 10^−5^	5.3 × 10^−5^	4.4 × 10^−5^	4.6 × 10^−5^
PFOA	192	191	118	107	126	146	121	129	192	191	118	107	126	146	121	129
PFOS	4.05	4.03	2.49	2.26	2.65	3.08	2.55	2.72	4.05	4.03	2.49	2.26	2.65	3.08	2.55	2.72
PFNA	4.74	4.72	2.91	2.65	3.10	3.60	2.98	3.18	4.74	4.72	2.91	2.65	3.10	3.60	2.98	3.18
PFDA	4.4 × 10^−4^	4.4 × 10^−4^	2.7 × 10^−4^	2.4 × 10^−4^	2.9 × 10^−4^	3.3 × 10^−4^	2.8 × 10^−4^	2.9 × 10^−4^	4.4 × 10^−4^	4.4 × 10^−4^	2.7 × 10^−4^	2.4 × 10^−4^	2.9 × 10^−4^	3.3 × 10^−4^	2.8 × 10^−4^	2.9 × 10^−4^
PFNuDA	1.7 × 10^−3^	1.7 × 10^−3^	1.1 × 10^−3^	9.6 × 10^−4^	1.1 × 10^−3^	1.3 × 10^−3^	1.1 × 10^−3^	1.2 × 10^−3^	1.7 × 10^−3^	1.7 × 10^−3^	1.1 × 10^−3^	9.6 × 10^−4^	1.1 × 10^−3^	1.3 × 10^−3^	1.1 × 10^−3^	1.2 × 10^−3^
PFBS	3.8 × 10^−3^	3.8 × 10^−3^	2.3 × 10^−3^	2.1 × 10^−3^	2.5 × 10^−3^	2.9 × 10^−3^	2.4 × 10^−3^	2.6 × 10^−3^	3.8 × 10^−3^	3.8 × 10^−3^	2.3 × 10^−3^	2.1 × 10^−3^	2.5 × 10^−3^	2.9 × 10^−3^	2.4 × 10^−3^	2.6 × 10^−3^
PFOSA	2.5 × 10^−1^	2.5 × 10^−1^	1.6 × 10^−1^	1.4 × 10^−1^	1.7 × 10^−1^	1.9 × 10^−1^	1.6 × 10^−1^	1.7 × 10^−1^	2.5 × 10^−1^	2.5 × 10^−1^	1.6 × 10^−1^	1.4 × 10^−1^	1.7 × 10^−1^	1.9 × 10^−1^	1.6 × 10^−1^	1.7 × 10^−1^
PFDS	6.5 × 10^−2^	6.5 × 10^−2^	4.0 × 10^−2^	3.7 × 10^−2^	4.3 × 10^−2^	5.0 × 10^−2^	4.1 × 10^−2^	4.4 × 10^−2^	6.5 × 10^−2^	6.5 × 10^−2^	4.0 × 10^−2^	3.7 × 10^−2^	4.3 × 10^−2^	5.0 × 10^−2^	4.1 × 10^−2^	4.4 × 10^−2^
PFDoDA	1.4 × 10^−1^	1.4 × 10^−1^	8.5 × 10^−2^	7.7 × 10^−2^	9.0 × 10^−2^	1.1 × 10^−1^	8.7 × 10^−2^	9.3 × 10^−2^	1.4 × 10^−1^	1.4 × 10^−1^	8.5 × 10^−2^	7.7 × 10^−2^	9.0 × 10^−2^	1.1 × 10^−1^	8.7 × 10^−2^	9.3 × 10^−2^
PFHxS	6.4 × 10^−1^	6.4 × 10^−1^	3.9 × 10^−1^	3.6 × 10^−1^	4.2 × 10^−1^	4.9 × 10^−1^	4.0 × 10^−1^	4.3 × 10^−1^	6.4 × 10^−1^	6.4 × 10^−1^	3.9 × 10^−1^	3.6 × 10^−1^	4.2 × 10^−1^	4.9 × 10^−1^	4.0 × 10^−1^	4.3 × 10^−1^

## Data Availability

Dataset available on request from the authors.

## Supplementary Materials

The following supporting information can be downloaded at: https://www.mdpi.com/article/10.3390/jox15030081/s1, Table S1: Phisico-chemical properties of PFAS compounds; Table S2: The gradient program used for the separation of the 29 compounds; Table S3: Acquisition windows set for the most sensitive detection of the analytes of interest; Figure S1. MRM chromatogram of PFAS mixture of 50 µg/L; Table S4: MRM transitions (Q—quatifier; q—qualifier) and MS operational parameters; Table S5: NOAEL values used for TDI calculation, except PFOS, PFOA, PFHxS, and PFNA and their corresponding TDI values, which are established by EFSA. Table S6: Concentration values (ng/L) of EU-regulated PFASs in bottled (commercial) water; Table S7: Concentration values (ng/L) of EU-regulated PFASs in tap water; Table S8: Concentration values (ng/L) of unregulated PFASs in bottled (commercial) water; Table S9: Concentration values (ng/L) of unregulated PFASs in tap water; Table S10: Drinking water consumption and average body weight for different age groups/sex; Table S11. EDI values were calculated using the values determined in bottled and tap water.
