# Supplementary material for: Unregulated and Regulated PFASs in Bottled and Tap Water: Occurrence, Co-Occurrence Patterns, and Implications for Human Health and Regulatory Frameworks
Source: J Xenobiot. 2025 May 27;15(3):81. doi: 10.3390/jox15030081 (PMC12194177; doi:10.3390/jox15030081)
Supplement: Supplementary file 1 [file jox-15-00081-s001.zip › jox-3584323-supplementary.pdf]

Unregulated and Regulated PFAS in Bottled and Tap Water: Occurrence, Co-Occurrence Patterns, and Implications for Hu-man Health and Regulatory Frameworks

Ioana-Antonia Cimpean, Iuliana Paun*, Florinela Pirvu, Vasile Ion Iancu, Florentina Laura Chiriac*

National Research and Development Institute for Industrial Ecology – ECOIND, Drumul Podu Dambovitei 57-73, Sector 6, 060652, Bucharest, Romania.

*Corresponding author e-mail: laura.chiriac@incdecoind.ro (F.L.C.); iuliana.paun@incdecoind.ro (V.I.I.)

Table S1. Physico-chemical properties of PFAS compounds

Nr. Crt.	Abreviere	Analiti	Formula moleculara	Masa moleculara	Log Kow (LogP)	Log Koc	LogD (pH 5.5)	LogD (pH 7.4)
1	PFBA	Perfluorobutanoic acid	$C_4HF_7O_2$	214	2.43	1.00	-1.11	-1.13
2	PFPeA	Perfluoropentanoicacid	$C_5HF_9O_2$	264	3.4	1.00	-0.32	-0.34
3	PFHxA	Perfluorohexanoic acid	$C_6HF_{11}O_2$	314	4.37	1.00	0.17	0.15
4	PFHpA	Perfluoroheptanoic acid	$C_7HF_{13}O_2$	364	5.33	1.97	1.14	1.11
5	PFOA	Perfluorooctanoic acid	$C_8HF_{15}O_2$	414	6.3	4.81	1.85	1.82
6	PFNA	Perfluorononanoic acid	$C_9HF_{17}O_2$	464	7.27	17.2	2.86	2.84
7	PFDA	Perfluorodecanoic acid	$C_{10}HF_{19}O_2$	514	9.53	45.8	3.64	3.62
8	PFUnA	Perfluoroundecanoic acid	$C_{11}HF_{21}O_2$	564	10.42	92.7	4.25	4.23
9	PFDoA	Perfluorododecanoic acid	$C_{12}HF_{23}O_2$	614	11.3	151	4.60	4.58
10	PFBS	Perfluorobutanesulfonic acid	$C_4HF_9O_3S$	300	1.82	1.93	-1.56	-1.56
11	PFHxS	Perfluorohexanesulfonic acid	$C_6HF_{13}O_3S$	399	5.25	1.00	-0.54	-0.54
12	PFHpS	Perfluoroheptanesulfonic acid	$C_7HF_{15}O_3S$	450	6.14	1.00	0.10	0.10
13	PFOS	Perflorooctanesulfonic acid	$C_8HF_{17}O_3S$	499	7.03	1.39	0.66	0.66
14	4:2 FTS	1H,1H,2H,2H-Perfluorohexanesulfonate	$C_6H_5F_9O_3S$	328	1.70	1.00	-2.27	-2.27
15	6:2 FTS	1H,1H,2H,2H-Perfluorooctanesulfonate	$C_8H_5F_{13}O_3S$	428	3.47	1.00	-0.92	-1.00
16	8:2 FTS	1H,1H,2H,2H-Perfluorodecanesulfonate	$C_{10}H_5F_{17}O_3S$	528	5.25	1.00	0.23	0.15
17	HFPO-DA (GenX)	2,3,3,3-Tetrafluoro-2-(heptafluoropropoxy)propionic acid	$C_6HF_{11}O_3$	330	8.12	2.48	1.34	1.34
18	DONA	Dodecafluoro-3H-4,8-dioxanonanoic acid	$C_7H_2F_{12}O_4$	378	8.65	6.33	2.06	2.04
19	9Cl-PF3ONS	9-Chlorohexadecafluoro-3-oxanone-1-sulfonic acid, potassium salt	$C_8ClF_{16}KO_4S$	570	-	-	-	-
20	11Cl-PF3OUdS	11-Chloroeicosafluoro-3-oxaundecane-1-sulfonic acid, potassium salt	$C_{10}ClF_{20}KO_4S$	670	-	-	-	-
21	PFMPA	Perfluoro-3-methoxypropanoic acid	$C_4HF_7O_3$	230	5.41	1.00	-0.55	-0.58
22	PFMBA	Perfluoro-4-methoxybutanoic acid	$C_5HF_9O_3$	280	6.42	1.00	0.27	0.24
23	PFDHA	Perfluoro-3,6-dioxaheptanoic acid	$C_5HF_9O_4$	296	7.69	2.74	1.41	1.40
24	PFEESA	Perfluoro(2-ethoxyethane) sulfonic acid	$C_4HF_9O_4S$	316	5.09	1.00	-0.92	-0.92
25	PFNS	Perfluorononanesulfonic acid	$C_9HF_{19}O_3S$	550	7.92	3.28	1.35	1.35
26	PFDS	Perfluorodecanesulfonic acid	$C_{10}HF_{21}O_3S$	600	8.81	8.65	2.13	2.13

27	PFOSA	Perfluorooctanesulfonamide	$C_8H_2F_{17}NO_2S$	499	7.64	2350<br>7	5.51	5.00
28	$^{13}C_3$-PFBA	$^{13}C_3$- perfluorobutanoic acid	$C_3F_7O_2 \cdot Na$	239	-	-	-	-
29	$^{13}C_8$-PFOA	$^{13}C_8$- perfluorooctanoic acid	$C_8F_{17}SO_2NH_2$	499	-	-	-	-

* Experimental values reported in international databases. https://pubchem.ncbi.nlm.nih.gov/

Table S2. The gradient program used for the separation of the 29 compounds.

	Time	A	B	Flow	Pressure
1	0.00 min	95.0 %	5.0 %	0.400 mL/min	400.00 bar
2	1.00 min	75.0 %	25.0 %	0.400 mL/min	400.00 bar
3	1.50 min	45.0 %	55.0 %	0.400 mL/min	400.00 bar
4	5.50 min	30.0 %	70.0 %	0.400 mL/min	400.00 bar
5	7.00 min	20.0 %	80.0 %	0.400 mL/min	400.00 bar
6	8.50 min	20.0 %	80.0 %	0.400 mL/min	400.00 bar
7	8.51 min	95.0 %	5.0 %	0.400 mL/min	400.00 bar

Table S3. Acquisition windows set for the most sensitive detection of the analytes of interest.

Index	Start Time (min)	Scan Type	Ion Mode	Div Valve	Store
1	0	MRM	ESI	To Waste	No
2	3.2	MRM	ESI	To MS	Yes
3	4.4	MRM	ESI	To MS	Yes
4	4.9	MRM	ESI	To MS	Yes
5	6.3	MRM	ESI	To MS	Yes
6	7.1	MRM	ESI	To MS	Yes
7	7.9	MRM	ESI	To MS	Yes
8	8.5	MRM	ESI	To MS	Yes
9	8.85	MRM	ESI	To MS	Yes
10	9.3	MRM	ESI	To MS	Yes
11	10.6	MRM	ESI	To Waste	No

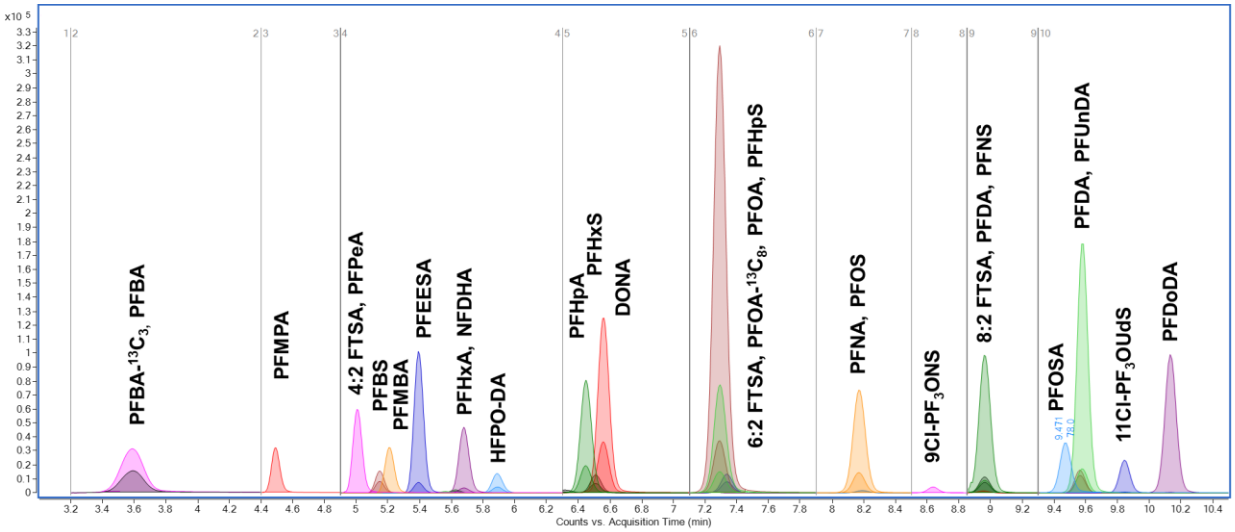

Figure S1. MRM chromatogram of PFAS mixture of 50 μg/L

Table S4. MRM transitions (Q - quatifier, q – qualifier) and MS operational parameters

PFAS	IS	Prec Ion	MS1 Res	Prod Ion	Q/q	MS2 Res	Dwell	Frag (V)	CE (V)	Cell Acc (V)	Polarity
PFBA-$^{13}C_3$	Yes	216	Unit	172	Q	Unit	250	80	2	5	Negative
PFBA	No	213	Unit	168.7	Q	Unit	250	60	4	1	Negative
PFMPA	No	229	Unit	85	Q	Unit	250	60	12	4	Negative
4:2 FTSA	No	327	Unit	307	Q	Unit	30	30	15	4	Negative
4:2 FTSA	No	327	Unit	80.9	q	Wide	30	90	45	6	Negative
PFEESA	No	315	Unit	135	Q	Unit	30	110	24	4	Negative
PFEESA	No	315	Unit	69	q	Wide	30	103	56	4	Negative
PFHxA	No	313	Unit	269	Q	Unit	30	80	3	6	Negative
PFHxA	No	313	Unit	119	q	Wide	30	70	18	6	Negative
PFBS	No	299	Unit	98.9	q	Wide	30	110	22	6	Negative
PFBS	No	299	Unit	80	Q	Unit	30	90	45	6	Negative
NFDHA	No	295	Unit	201	Q	Unit	30	75	10	4	Negative
NFDHA	No	295	Unit	85	q	Wide	30	83	40	4	Negative
HFPO-DA	No	285	Unit	185	q	Wide	30	80	4	4	Negative
HFPO-DA	No	285	Unit	169	Q	Unit	30	75	4	4	Negative
PFMBA	No	279	Unit	235	q	Wide	30	80	2	4	Negative
PFMBA	No	279	Unit	85	Q	Unit	30	70	12	4	Negative
PFPeA	No	263	Unit	219	Q	Unit	30	71	3	5	Negative
PFHxS	No	398.9	Unit	99	q	Wide	80	100	50	4	Negative
PFHxS	No	398.9	Unit	80	Q	Unit	80	120	50	4	Negative
DONA	No	377	Unit	251	Q	Unit	80	90	5	4	Negative
DONA	No	377	Unit	85	q	Unit	80	90	35	4	Negative
PFHpA	No	363	Unit	319	Q	Unit	80	75	1	4	Negative
PFHpA	No	363	Unit	169	q	Wide	80	75	12	4	Negative
PFHpS	No	449	Unit	99	q	Wide	60	135	50	4	Negative
PFHpS	No	449	Unit	80	Q	Unit	60	135	50	4	Negative
6:2 FTSA	No	427	Unit	406.8	q	Unit	60	60	10	4	Negative
6:2 FTSA	No	427	Unit	80.9	Q	Wide	60	60	20	4	Negative
PFOA $^{13}C_8$	Yes	421	Unit	376	Q	Unit	60	83	4	3	Negative
PFOA $^{13}C_8$	Yes	421	Unit	172	q	Wide	60	83	8	3	Negative
PFOA	No	413	Unit	369	Q	Unit	60	90	1	2	Negative
PFOA	No	413	Unit	169	q	Unit	60	90	10	2	Negative
PFOS	No	499	Unit	99	Q	Wide	120	140	30	5	Negative
PFOS	No	499	Unit	80	q	Unit	120	140	30	5	Negative
PFNA	No	462.9	Unit	418.9	Q	Unit	120	75	2	1	Negative
PFNA	No	462.9	Unit	169	q	Wide	120	75	15	1	Negative
9Cl-PF_3ONS	No	531	Unit	351	Q	Unit	250	150	10	4	Negative
PFNS	No	549	Unit	99	q	Wide	80	135	60	6	Negative
PFNS	No	549	Unit	80	Q	Unit	80	135	60	6	Negative
8:2 FTSA	No	527	Unit	506.8	Q	Wide	80	173	40	5	Negative
8:2 FTSA	No	527	Unit	81	q	Unit	80	90	40	5	Negative
PFDA	No	513	Unit	468.6	Q	Unit	80	90	10	1	Negative
PFDA	No	513	Unit	218.7	q	Wide	80	80	10	4	Negative
11Cl-PF_3OUdS	No	631	Unit	451	Q	Unit	70	60	20	4	Negative
11Cl-PF_3OUdS	No	631	Unit	83	q	Wide	70	60	20	4	Negative
PFDoDA	No	613	Unit	568.9	Q	Unit	70	30	10	4	Negative
PFDoDA	No	613	Unit	268.9	q	Wide	70	135	1	4	Negative
PFDS	No	599	Unit	99	q	Wide	70	135	60	6	Negative
PFDS	No	599	Unit	80	Q	Unit	70	135	60	6	Negative
PFUnDA	No	563	Unit	519	Q	Unit	70	80	10	1	Negative
PFUnDA	No	563	Unit	269	q	Unit	70	80	20	4	Negative
PFOSA	No	498	Unit	78	Q	Unit	70	135	45	5	Negative

Table S5. NOAELvalues used for TDI calculation, except PFOS, PFOA, PFHxS and PFNA and their corresponding TDI values, which are established by EFSA.

PFAS	NOAEL (mg/Kg bw/day)	TDI (ng/Kg bw/day)
PFBA	3.01	15050
PFHxA	20	100000
PFHpA	20	100000
PFOA		**0.63**
PFOS		**0.63**
PFNA	0.83	**0.63**
PFHxS		**0.63**
PFDA	1.20	6000
PFNuDA	1.01	5050
PFBS	1.00	5000
PFOSA	0.02	120
PFDS	0.03	145.00
PFDoDA	0.02	100.00

*TDI established by EFSA: TWI 4.4 ng/Kg body sum of PFOA, PFNA, PFOS and PFHxS

Table S6. Concentration values (ng/L) of EU regulated PFAS in bottled (commercial) waters

Samples	PFBA	PFBS	PFHxA	PFPeA	PFHpA	PFHxS	PFHpS	PFOA	PFNA	PFOS	PFDA	PFNS	PFDoDA	PFDS	PFOSA	PFUnDA
CW-1	0.06	<LOQ	<LOQ	<LOQ	<LOQ	<LOQ	4.15	<LOQ	<LOQ	<LOQ	<LOQ	<LOQ	0.06	<LOQ	0.90	0.16
CW-2	0.22	<LOQ	0.19	0.37	0.22	<LOQ	0.18	4.31	0.07	<LOQ	<LOQ	<LOQ	<LOQ	<LOQ	0.06	0.08
CW-3	0.09	<LOQ	<LOQ	0.25	0.25	<LOQ	0.22	3.94	<LOQ	<LOQ	<LOQ	<LOQ	<LOQ	<LOQ	0.07	0.12
CW-4	0.34	<LOQ	0.29	0.20	0.24	<LOQ	<LOQ	4.09	<LOQ	<LOQ	<LOQ	<LOQ	<LOQ	<LOQ	1.13	0.10
CW-5	0.08	<LOQ	0.26	0.24	0.21	<LOQ	0.22	4.18	<LOQ	<LOQ	<LOQ	<LOQ	0.07	<LOQ	0.78	0.10
CW-6	<LOQ	<LOQ	0.24	0.10	0.19	<LOQ	<LOQ	4.24	<LOQ	<LOQ	0.06	<LOQ	<LOQ	<LOQ	<LOQ	0.10
CW-7	<LOQ	<LOQ	<LOQ	0.06	0.21	<LOQ	<LOQ	4.35	0.07	<LOQ	<LOQ	<LOQ	0.11	<LOQ	0.18	0.15
CW-8	<LOQ	<LOQ	0.29	0.22	0.21	<LOQ	0.16	3.87	<LOQ	<LOQ	<LOQ	<LOQ	<LOQ	<LOQ	<LOQ	0.08
CW-9	<LOQ	<LOQ	0.26	0.21	0.20	<LOQ	0.21	3.87	0.07	<LOQ	<LOQ	<LOQ	0.07	<LOQ	<LOQ	0.08
CW-10	<LOQ	<LOQ	0.23	0.39	0.20	<LOQ	<LOQ	4.04	0.07	<LOQ	0.07	<LOQ	0.15	0.08	0.09	0.11
CW-11	<LOQ	<LOQ	0.24	0.24	0.25	<LOQ	0.08	4.27	<LOQ	<LOQ	0.10	0.13	0.51	0.28	0.11	0.32
CW-12	<LOQ	<LOQ	0.19	0.08	0.19	<LOQ	0.13	4.20	<LOQ	<LOQ	<LOQ	<LOQ	<LOQ	<LOQ	<LOQ	<LOQ
CW-13	<LOQ	<LOQ	0.26	<LOQ	0.23	<LOQ	<LOQ	4.03	<LOQ	<LOQ	<LOQ	<LOQ	0.28	0.12	0.16	0.09
CW-14	0.18	<LOQ	0.29	0.15	0.21	<LOQ	0.23	4.04	0.06	<LOQ	<LOQ	<LOQ	<LOQ	<LOQ	<LOQ	0.08
CW-15	<LOQ	<LOQ	0.22	0.42	0.19	<LOQ	<LOQ	3.87	<LOQ	<LOQ	<LOQ	<LOQ	<LOQ	<LOQ	<LOQ	<LOQ
CW-16	<LOQ	<LOQ	0.25	0.20	0.20	<LOQ	<LOQ	4.48	0.11	0.09	0.07	0.06	0.22	0.11	0.15	0.13
CW-17	<LOQ	<LOQ	0.23	<LOQ	0.26	<LOQ	0.16	3.99	0.08	<LOQ	<LOQ	0.39	<LOQ	0.35	0.94	<LOQ
CW-18	0.07	<LOQ	0.26	0.16	0.23	<LOQ	0.16	3.74	<LOQ	<LOQ	<LOQ	<LOQ	<LOQ	<LOQ	<LOQ	0.08
CW-19	<LOQ	0.70	0.23	0.37	0.19	<LOQ	0.23	3.78	0.07	<LOQ	0.06	<LOQ	<LOQ	<LOQ	<LOQ	<LOQ
CW-20	<LOQ	<LOQ	0.18	0.20	0.22	<LOQ	0.15	3.77	<LOQ	<LOQ	<LOQ	<LOQ	<LOQ	<LOQ	<LOQ	0.09
CW-21	<LOQ	<LOQ	0.25	0.07	0.21	<LOQ	0.17	3.78	<LOQ	<LOQ	<LOQ	<LOQ	<LOQ	<LOQ	<LOQ	<LOQ

Table S7. Concentration values (ng/L) of EU regulated PFAS in tap waters

Tap water	PFBA	PFBS	PFHxA	PFPeA	PFHpA	PFHxS	PFHpS	PFOA	PFNA	PFOS	PFDA	PFNS	PFDoDA	PFDS	PFOSA	PFUnDA
TW-1	0.48	<LOQ	0.18	0.36	0.27	<LOQ	0.14	4.84	0.13	<LOQ	<LOQ	<LOQ	0.24	0.17	0.08	0.14
TW-2	0.23	1.28	0.67	0.74	0.48	0.12	0.18	5.67	0.11	0.28	<LOQ	<LOQ	0.14	0.08	<LOQ	0.06
TW-3	0.21	0.07	0.24	0.15	0.27	<LOQ	0.20	4.95	0.07	0.09	<LOQ	0.06	0.12	0.09	<LOQ	<LOQ
TW-4	<LOQ	0.91	0.48	0.17	0.40	0.06	0.15	8.57	<LOQ	0.22	<LOQ	0.08	0.17	0.11	<LOQ	0.11
TW-5	<LOQ	<LOQ	<LOQ	0.26	<LOQ	<LOQ	<LOQ	1.88	0.11	<LOQ	<LOQ	<LOQ	0.22	<LOQ	<LOQ	0.11
TW-6	0.08	0.06	0.26	<LOQ	0.19	<LOQ	0.25	8.80	0.09	<LOQ	<LOQ	<LOQ	0.21	<LOQ	<LOQ	<LOQ
TW-7	0.08	<LOQ	<LOQ	0.19	0.21	<LOQ	0.17	9.61	0.15	0.08	<LOQ	0.09	0.22	<LOQ	<LOQ	0.10
TW-8	<LOQ	0.90	0.40	0.31	0.39	<LOQ	0.17	12.74	0.09	0.20	<LOQ	<LOQ	0.20	0.06	<LOQ	0.08
TW-9	0.11	1.02	0.41	<LOQ	0.42	0.09	0.16	12.38	0.12	<LOQ	<LOQ	0.08	0.21	<LOQ	<LOQ	0.12
TW-10	0.13	<LOQ	0.23	0.14	0.32	<LOQ	0.29	14.82	0.14	<LOQ	<LOQ	<LOQ	0.22	0.08	0.06	0.17
TW-11	0.23	<LOQ	0.27	0.10	0.29	<LOQ	0.30	15.11	0.12	0.11	0.07	0.09	0.23	0.12	<LOQ	<LOQ
TW-12	0.10	<LOQ	0.18	0.26	0.37	<LOQ	0.09	13.59	0.11	<LOQ	0.08	<LOQ	0.19	0.07	0.07	<LOQ
TW-13	0.21	<LOQ	0.23	0.26	0.34	<LOQ	0.18	12.15	0.14	<LOQ	<LOQ	<LOQ	0.25	<LOQ	0.06	0.07
TW-14	0.19	<LOQ	0.27	0.26	0.40	<LOQ	0.22	17.16	0.13	<LOQ	0.06	0.09	0.28	<LOQ	<LOQ	0.12
TW-15	<LOQ	<LOQ	0.26	0.29	0.35	<LOQ	0.27	12.43	0.17	<LOQ	<LOQ	0.09	0.25	0.08	<LOQ	0.18
TW-16	1.96	<LOQ	0.28	0.07	0.21	<LOQ	0.26	4.71	0.09	0.37	0.12	0.07	0.17	<LOQ	<LOQ	0.18
TW-17	0.28	0.80	0.44	0.35	0.53	<LOQ	<LOQ	6.55	0.14	0.45	0.12	0.08	0.19	<LOQ	<LOQ	0.10
TW-18	0.73	0.72	0.36	0.37	0.50	<LOQ	0.27	6.26	0.14	0.26	0.08	<LOQ	0.20	<LOQ	0.06	0.09
TW-19	0.89	1.62	0.88	1.08	1.16	0.13	0.93	6.78	0.85	<LOQ	1.69	<LOQ	<LOQ	<LOQ	<LOQ	<LOQ
TW-20	<LOQ	0.12	<LOQ	<LOQ	<LOQ	<LOQ	<LOQ	4.84	0.08	<LOQ	<LOQ	0.07	<LOQ	<LOQ	<LOQ	0.07
TW-21	0.58	<LOQ	0.09	0.31	0.20	<LOQ	0.16	4.60	0.07	<LOQ	<LOQ	<LOQ	0.09	<LOQ	<LOQ	0.09
TW-22	0.21	<LOQ	0.15	<LOQ	0.17	<LOQ	0.26	4.86	0.09	0.08	<LOQ	<LOQ	0.10	<LOQ	<LOQ	0.08
TW-23	0.59	<LOQ	0.14	0.19	0.14	<LOQ	<LOQ	4.78	0.09	<LOQ	<LOQ	<LOQ	<LOQ	0.08	<LOQ	0.08
TW-24	<LOQ	0.09	<LOQ	<LOQ	<LOQ	<LOQ	0.25	5.32	0.08	0.14	<LOQ	<LOQ	<LOQ	<LOQ	<LOQ	<LOQ

Table S8. Concentration values (ng/L) of un-regulated PFAS in bottled (commercial) waters

Bottled Water	PFMPA	4:2 FTSA	HFPO-DA	NFDHA	PFEESA	PFMBA	DONA	6:2 FTSA	9Cl-PF3ONS	8:2 FTSA	11Cl-PF3OUdS	PFOSA
CW-1	<LOQ	0.689	<LOQ	0.075	0.136	0.167	0.147	4.636	<LOQ	0.064	<LOQ	0.899
CW-2	<LOQ	<LOQ	<LOQ	0.113	<LOQ	<LOQ	<LOQ	<LOQ	<LOQ	<LOQ	<LOQ	0.062
CW-3	<LOQ	<LOQ	<LOQ	0.148	<LOQ	<LOQ	<LOQ	<LOQ	<LOQ	<LOQ	<LOQ	0.074
CW-4	<LOQ	<LOQ	0.130	<LOQ	<LOQ	<LOQ	<LOQ	0.135	<LOQ	<LOQ	<LOQ	1.131
CW-5	<LOQ	<LOQ	0.067	<LOQ	<LOQ	<LOQ	<LOQ	0.176	<LOQ	<LOQ	<LOQ	0.781
CW-6	<LOQ	<LOQ	<LOQ	0.172	<LOQ	<LOQ	<LOQ	<LOQ	<LOQ	<LOQ	<LOQ	<LOQ
CW-7	0.545	<LOQ	0.135	0.109	<LOQ	<LOQ	<LOQ	0.146	<LOQ	<LOQ	<LOQ	0.175
CW-8	<LOQ	<LOQ	0.213	0.206	<LOQ	<LOQ	<LOQ	0.146	<LOQ	<LOQ	<LOQ	<LOQ
CW-9	<LOQ	<LOQ	<LOQ	<LOQ	<LOQ	<LOQ	<LOQ	0.084	<LOQ	<LOQ	<LOQ	<LOQ
CW-10	<LOQ	<LOQ	<LOQ	0.148	<LOQ	<LOQ	<LOQ	0.208	<LOQ	<LOQ	<LOQ	0.095
CW-11	0.078	0.062	<LOQ	0.070	<LOQ	<LOQ	<LOQ	0.063	<LOQ	0.199	0.298	0.111
CW-12	<LOQ	<LOQ	0.168	0.092	<LOQ	<LOQ	<LOQ	0.160	<LOQ	<LOQ	<LOQ	<LOQ
CW-13	0.221	<LOQ	<LOQ	<LOQ	<LOQ	<LOQ	<LOQ	<LOQ	<LOQ	<LOQ	0.108	0.159
CW-14	0.165	<LOQ	<LOQ	0.100	<LOQ	<LOQ	<LOQ	0.069	<LOQ	<LOQ	<LOQ	<LOQ
CW-15	0.100	<LOQ	<LOQ	0.090	<LOQ	<LOQ	<LOQ	<LOQ	<LOQ	<LOQ	<LOQ	<LOQ
CW-16	0.118	0.081	0.114	0.108	<LOQ	<LOQ	<LOQ	0.214	<LOQ	<LOQ	0.148	0.153
CW-17	0.065	<LOQ	<LOQ	0.234	<LOQ	<LOQ	<LOQ	0.258	0.062	1.467	0.785	0.940
CW-18	<LOQ	<LOQ	<LOQ	0.070	<LOQ	<LOQ	<LOQ	0.258	<LOQ	<LOQ	<LOQ	<LOQ
CW-19	<LOQ	<LOQ	<LOQ	0.083	<LOQ	<LOQ	<LOQ	<LOQ	<LOQ	<LOQ	<LOQ	<LOQ
CW-20	<LOQ	<LOQ	<LOQ	0.063	<LOQ	<LOQ	<LOQ	0.142	<LOQ	<LOQ	<LOQ	<LOQ
CW-21	0.063	<LOQ	0.106	0.075	<LOQ	<LOQ	<LOQ	0.284	<LOQ	<LOQ	<LOQ	<LOQ

Table S9. Concentration values (ng/L) of un-regulated PFAS in tap waters

Tap water	PFMPA	4:2 FTSA	HFPO-DA	NFDHA	PFEESA	PFMBA	DONA	6:2 FTSA	9Cl-PF3ONS	8:2 FTSA	11Cl-PF3OUdS	PFOSA
TW-1	0.28	<LOQ	0.06	0.22	<LOQ	<LOQ	0.13	0.16	<LOQ	<LOQ	0.13	0.08
TW-2	0.17	<LOQ	0.23	0.10	<LOQ	0.07	0.09	0.27	<LOQ	<LOQ	0.06	<LOQ
TW-3	0.14	0.07	<LOQ	0.15	<LOQ	0.08	0.10	0.10	<LOQ	<LOQ	0.09	<LOQ
TW-4	0.08	<LOQ	<LOQ	0.36	<LOQ	<LOQ	<LOQ	0.14	<LOQ	<LOQ	0.09	<LOQ
TW-5	<LOQ	0.07	0.07	0.18	<LOQ	<LOQ	<LOQ	<LOQ	<LOQ	<LOQ	<LOQ	<LOQ
TW-6	<LOQ	<LOQ	<LOQ	0.12	<LOQ	<LOQ	0.08	0.09	<LOQ	<LOQ	<LOQ	<LOQ
TW-7	<LOQ	<LOQ	<LOQ	0.08	<LOQ	<LOQ	0.09	0.07	<LOQ	0.08	<LOQ	<LOQ
TW-8	0.09	<LOQ	<LOQ	<LOQ	<LOQ	<LOQ	<LOQ	0.29	0.10	<LOQ	0.07	<LOQ
TW-9	<LOQ	<LOQ	<LOQ	0.08	<LOQ	<LOQ	<LOQ	0.65	<LOQ	<LOQ	<LOQ	<LOQ
TW-10	0.36	<LOQ	0.08	0.08	<LOQ	<LOQ	0.11	0.21	<LOQ	<LOQ	<LOQ	0.06
TW-11	0.12	<LOQ	0.09	0.16	<LOQ	<LOQ	0.12	<LOQ	<LOQ	<LOQ	<LOQ	<LOQ
TW-12	0.09	0.12	0.06	0.08	<LOQ	<LOQ	0.07	0.16	<LOQ	<LOQ	<LOQ	0.07
TW-13	0.10	<LOQ	<LOQ	0.07	<LOQ	<LOQ	0.08	0.28	<LOQ	<LOQ	<LOQ	0.06
TW-14	0.09	<LOQ	<LOQ	<LOQ	<LOQ	<LOQ	<LOQ	<LOQ	<LOQ	<LOQ	<LOQ	<LOQ
TW-15	0.13	<LOQ	<LOQ	0.23	<LOQ	<LOQ	0.06	0.20	<LOQ	<LOQ	<LOQ	<LOQ
TW-16	0.14	<LOQ	0.07	0.12	<LOQ	0.07	<LOQ	0.11	<LOQ	0.16	<LOQ	<LOQ
TW-17	0.20	0.15	0.08	0.24	<LOQ	0.09	0.06	0.06	<LOQ	0.16	<LOQ	<LOQ
TW-18	0.19	<LOQ	0.09	0.16	<LOQ	<LOQ	<LOQ	0.37	<LOQ	<LOQ	<LOQ	0.06
TW-19	0.19	2.02	0.50	0.31	0.58	0.58	0.67	2.12	<LOQ	0.30	<LOQ	<LOQ
TW-20	<LOQ	0.29	<LOQ	0.08	0.10	<LOQ	0.09	0.35	<LOQ	0.09	<LOQ	<LOQ
TW-21	<LOQ	<LOQ	0.10	0.12	<LOQ	<LOQ	<LOQ	0.46	<LOQ	0.13	<LOQ	<LOQ
TW-22	<LOQ	<LOQ	0.11	0.09	<LOQ	0.08	<LOQ	0.46	<LOQ	<LOQ	<LOQ	<LOQ
TW-23	<LOQ	<LOQ	0.09	0.10	<LOQ	0.07	<LOQ	0.16	<LOQ	<LOQ	<LOQ	<LOQ
TW-24	<LOQ	0.38	<LOQ	0.23	0.10	0.18	0.09	<LOQ	<LOQ	0.06	<LOQ	<LOQ

Table S10. Drinking water consumption and average body weight for different age groups/sex

Age categories	Sex	Body weight (Kg)	Water (mL)
Kids (6-11 years)	Masculine	31	838
	Feminine	26	700
Teenagers (12-19 years)	Masculine	56	929
	Feminine	53	800
Adults (20-60 years)	Masculine	70	1236
	Feminine	55	1130
Seniors (>60 years)	Masculine	65	1106
	Feminine	60	1090

Table S11. EDI values were calculated using the values determined in bottled and tap water

PFAS	EDI bottled water (ng/Kg bw/day)								EDI tap water (ng/Kg bw/day)							
	Kids (6-11 years)		Teenagers (12-19 years)		Adults (20-60 years)		Seniors (>60 years)		Kids (6-11 years)		Teenagers (12-19 years)		Adults (20-60 years)		Seniors (>60 years)	
	M	F	M	F	M	F	M	F	M	F	M	F	M	F	M	F
PFBA	9.15	9.11	5.61	5.11	5.97	6.95	5.76	6.15	53.1	52.9	32.6	29.6	34.7	40.3	33.4	35.7
PFPeA	11.4	11.3	6.98	6.35	7.43	8.65	7.16	7.65	29.2	29.1	17.9	16.3	19.1	22.2	18.4	19.6
PFHxA	7.93	7.90	4.87	4.43	5.18	6.03	4.99	5.33	23.8	23.7	14.6	13.3	15.6	18.1	15.0	16.0
PFHpA	6.91	6.88	4.24	3.86	4.52	5.25	4.35	4.65	31.4	31.3	19.3	17.5	20.5	23.9	19.8	21.1
PFOA	121	121	74.3	67.6	79.1	92.0	76.2	81.4	464	462	285	259	303	353	292	312
PFOS	2.55	2.54	1.57	1.42	1.67	1.94	1.61	1.71	46.8	46.6	28.7	26.1	30.6	35.6	29.4	31.4
PFNA	2.99	2.97	1.83	1.67	1.95	2.27	1.88	2.01	23.0	23.0	14.1	12.9	15.1	17.5	14.5	15.5
PFDA	2.63	2.62	1.61	1.47	1.72	2.00	1.66	1.77	45.6	45.4	28.0	25.5	29.8	34.7	28.7	30.6
PFNuDA	8.71	8.67	5.34	4.86	5.69	6.62	5.48	5.85	145	144	89.0	81.0	94.7	110.2	91.3	97.5
PFBS	19.0	19.0	11.7	10.6	12.4	14.5	11.9	12.8	43.8	43.6	26.9	24.4	28.6	33.3	27.5	29.4
PFOSA	30.6	30.4	18.8	17.1	20.0	23.2	19.2	20.5	62.5	62.2	38.3	34.9	40.8	47.5	39.3	42.0
PFDS	9.48	9.44	5.82	5.29	6.19	7.21	5.97	6.37	238	237	146	133	155	181	150	160
PFDoDA	13.8	13.8	8.48	7.72	9.03	10.51	8.70	9.29	194	193	119	108	126	147	122	130
PFHxS	0.40	0.40	0.25	0.23	0.26	0.31	0.25	0.27	3.48	3.46	2.13	1.94	2.27	2.64	2.19	2.34
